# Molecular Landscape of Pelvic Organ Prolapse Provides Insights into Disease Etiology

**DOI:** 10.3390/ijms24076087

**Published:** 2023-03-23

**Authors:** Kirsten B. Kluivers, Sabrina L. Lince, Alejandra M. Ruiz-Zapata, Wilke M. Post, Rufus Cartwright, Manon H. Kerkhof, Joanna Widomska, Ward De Witte, Jakub Pecanka, Lambertus A. Kiemeney, Sita H. Vermeulen, Jelle J. Goeman, Kristina Allen-Brady, Egbert Oosterwijk, Geert Poelmans

**Affiliations:** 1 Department of Obstetrics and Gynecology, Radboud Institute for Molecular Life Sciences, Radboud University Medical Center, 6525 GA Nijmegen, The Netherlands; kirsten.kluivers@radboudumc.nl (K.B.K.);; 2 Department of Urology, Radboud Institute for Molecular Life Sciences, Radboud University Medical Center, 6525 GA Nijmegen, The Netherlands; 3 Department of Gynaecology, Chelsea and Westminster NHS Foundation Trust, Department of Epidemiology and Biostatistics, Imperial College London, London SW7 2AZ, UK; 4 Department of Gynaecology and Reconstructive Pelvic Surgery, Curilion Women’s Health Clinic, 2015 BJ Haarlem, The Netherlands; 5 Department of Cognitive Neuroscience, Donders Institute for Brain, Cognition and Behaviour, Radboud University Medical Center, 6525 GD Nijmegen, The Netherlands; 6 Department of Human Genetics, Radboud University Medical Center, 6500 HB Nijmegen, The Netherlands; 7 Department of Medical Statistics and Bioinformatics, Leiden University Medical Center, 2333 ZA Leiden, The Netherlands; 8 Department for Health Evidence, Radboud Institute for Health Sciences, Radboud University Medical Center, 6525 EZ Nijmegen, The Netherlands; 9 Department of Internal Medicine, Genetic Epidemiology, University of Utah, Salt Lake City, UT 84132, USA

**Keywords:** pelvic organ prolapse, exome chip study, genetics, single nucleotide variants (SNVs), TGFB1, metformin

## Abstract

Pelvic organ prolapse (POP) represents a major health care burden in women, but its underlying pathophysiological mechanisms have not been elucidated. We first used a case-control design to perform an exome chip study in 526 women with POP and 960 control women to identify single nucleotide variants (SNVs) associated with the disease. We then integrated the functional interactions between the POP candidate proteins derived from the exome chip study and other POP candidate molecules into a molecular landscape. We found significant associations between POP and SNVs in 54 genes. The proteins encoded by 26 of these genes fit into the molecular landscape, together with 43 other POP candidate molecules. The POP landscape is located in and around epithelial cells and fibroblasts of the urogenital tract and harbors four interacting biological processes—epithelial-mesenchymal transition, immune response, modulation of the extracellular matrix, and fibroblast function—that are regulated by sex hormones and TGFB1. Our findings were corroborated by enrichment analyses of differential gene expression data from an independent POP cohort. Lastly, based on the landscape and using vaginal fibroblasts from women with POP, we predicted and showed that metformin alters gene expression in these fibroblasts in a beneficial direction. In conclusion, our integrated molecular landscape of POP provides insights into the biological processes underlying the disease and clues towards novel treatments.

## 1. Introduction

Pelvic organ prolapse (POP) represents a major health care burden in women, with a reported prevalence of up to 40% [1]. POP is not life-threatening, but its bothersome symptoms have a significant impact on quality of life. The cumulative life-time risk for a surgical intervention for POP is approximately 13% [2], and the total costs for POP surgery are substantial [3]. POP has a multifactorial etiology in which both hereditary and environmental factors play a role. Parity, vaginal delivery, birth weight, age, and high body mass index (BMI) have repeatedly been identified as environmental risk factors [4,5] and could all cause defects of pelvic floor tissues. Familial susceptibility to these defects seems likely, since women with POP more often have family members with POP compared to women without POP [6,7,8]. Evidence for a genetic background of POP has also emerged from twin studies, with approximately 40% of the POP liability being explained by genetic factors [9,10]. Candidate gene association studies have found a number of single nucleotide polymorphisms (SNPs) to be putatively associated with POP, including genes encoding steroid hormone receptors, collagens type I and III, and multiple matrix metalloproteinases. However, only one SNP, rs1800012 in the *COL1A1* gene, has demonstrated a replicable association with POP [11]. Thus far, two genome-wide association studies (GWASs) of POP have shown significant associations with SNPs. The first GWAS identified five SNPs associated with POP that were nominally replicated [12]. In 2020, the results of a second GWAS were reported, in which the authors found eight SNPs involving seven genes to be associated with POP at a genome-wide significant level (*p* < 5.00 × 10^−8^) [13]. As is the case in many other fields of medicine, the translational step from genetic data towards clinically useful information—i.e., diagnostic biomarkers and disease-modifying treatments—still needs to be made in the field of POP. Therefore, in this study, we conducted the first exome chip study of POP—which suggested multiple novel candidate genes—and integrated the exome chip results with other available genetic and expression data to generate and corroborate a molecular landscape of POP. This landscape provides detailed, novel insights into the biological processes that underpin pelvic floor function and dysfunction.

## 2. Results

### 2.1. Exome Chip Study

We conducted an exome chip study with 526 POP cases and 960 control women (without POP). The characteristics of all participants in the exome chip study are presented in Table 1. Compared to cases, controls were on average 5 years older and had a higher ratio of postmenopausal females. Since the prevalence of POP increases with age and is probably associated with postmenopausal state, this means that there would likely have been more POP-associated SNVs identified if the case group would have had a similar average age and a similar percentage of postmenopausal women than the control group. Therefore, the results of our study may be underestimating the real genetic differences. Since the prevalence of POP increases with age and is probably associated with a postmenopausal state, the results of our study may be underestimating the real genetic differences. We identified 54 statistically significant—i.e., with Bonferroni-corrected *p* < 0.05, uncorrected *p* < 1.48 × 10^−6^—exonic and (potentially) deleterious single nucleotide variants (SNVs), including 52 missense mutations, 1 splice site mutation, and 1 stop-gain mutation. The list of these SNVs and the genes they affect is provided in Table 2. For all 54 SNVs, a combined annotation-dependent depletion (CADD) score was available—a single pathogenicity score that combines 63 distinct computational prediction methods [14], including the Grantham score for missense mutations [15]—and 18 SNVs had a CADD score > 20, while 12 had a CADD score > 10 but < 20 (Table 2). These SNVs are predicted to be among the 1 and 10% most deleterious of all possible substitutions in the human genome, respectively [14,16]. This implies that a considerable proportion of the SNVs that we identified have a damaging effect on protein function.

### 2.2. Molecular Landscape of POP

**Figure 1:**
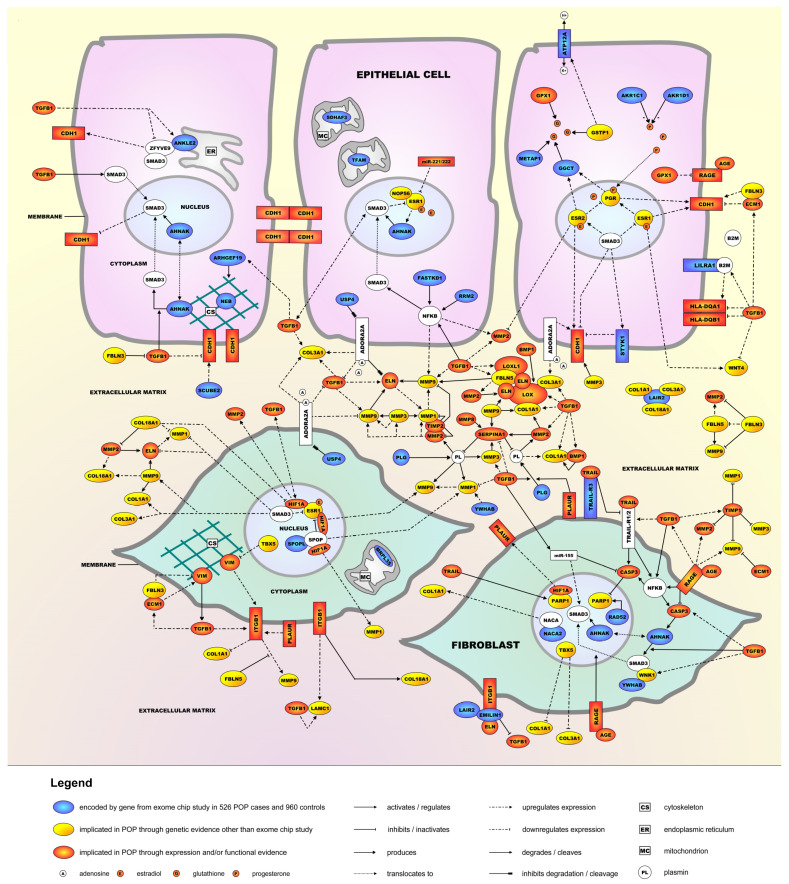
Molecular landscape of pelvic organ prolapse (POP). The POP landscape is located in and around epithelial cells and fibroblasts of the female urogenital tract. Four main biological processes operate in the landscape: (1) The first and most important process in the landscape is EMT. Under normal conditions, epithelial cells are connected to their environment through cell-cell interactions as well as cell-ECM interactions. Among the cell-cell interactions are epithelial cadherin (E-cadherin/CDH1)-based adherens junctions that stabilize epithelial cell-cell adhesion. EMT is characterized by a ‘cadherin switch’, which is initiated when epithelial cells start producing less CDH1 and more of the mesenchymal marker N-cadherin (CDH2) (not shown). This results in loss of epithelial cell-cell adhesion and the epithelial cells gradually transforming into mesenchymal cells, which then further differentiate into fibroblasts. EMT involves the key transcription factor SMAD3 and is negatively regulated by three sex hormone-bound receptors—the estrogen receptors ESR1 and ESR2 and the progesterone receptor (PGR)—that each upregulate CDH1 expression and hence prevent EMT. Conversely, TGFB1 induces EMT as it downregulates CDH1 expression. (2) Proteins of the major histocompatibility complex II (MHC II)—i.e., HLA-DQA1 and HLA-DQB1—are expressed in epithelial cells of the female reproductive tract, where they are involved in regulating the activity of the immune response through presenting foreign antigens to circulating T lymphocytes (not shown). TGFB1 regulates the expression of these two HLA proteins, in part through upregulating the expression of beta-2-microglobulin (B2M), an EMT-inducing extracellular protein that mediates antigen presentation. (3) Further, the ECM provides support to epithelial cells and fibroblasts, and modulation of the ECM is essential for many pathophysiological processes such as tissue growth, wound healing, and fibrosis. The ECM is composed of different molecules, including elastin (ELN) and collagen (COL) fibers, as well as proteins that crosslink or regulate the degradation of ELN and COL (e.g., LOX and LOXL1), various matrix metalloproteinases (MMPs) and their regulators, TIMP1 and TIMP2. Other important ECM proteins are ECM1, FBLN5, LAIR2, and LAMC1. TGFB1 is involved in ECM remodeling through regulating the expression of most ECM proteins in the landscape. (4) Since fibroblasts are responsible for the synthesis and secretion of the main ECM components through SMAD3—which, as indicated above, also plays a major role in EMT—fibroblast survival and apoptosis (and hence proper functioning) is another important landscape process. TRAIL, a cytokine of the tumor necrosis factor (TNF) family, regulates fibroblast survival and apoptosis through its receptors and signaling involving pro-apoptotic proteins such as NFKB and CASP3. As with the three other main processes, TGFB1 is a key regulator, e.g., through regulating the expression of TRAIL-R1 and CASP3 and activating NFKB. In addition, extracellular AGE (or advanced glycation end product) molecules binding to their receptor RAGE results in NFKB activation, CASP3 inhibition, and translocation of SMAD3 to the nucleus, which results in the AGE-RAGE complex stimulating the apoptosis of fibroblasts. Lastly, nuclear proteins such as HIF1A and PARP1 are also important regulators of fibroblast survival and apoptosis.

A molecular landscape was built in which 26 of the 54 proteins encoded by the genes from the exome chip study (Table 2) interact functionally, together with 41 other POP candidates as well as 2 POP-implicated microRNAs (Supplementary Table S1). The molecular landscape of POP is shown in Figure 1 and includes 10 proteins/molecules (indicated in white) that have not been directly linked to POP (yet) but show important functional interactions within the landscape.

The molecular POP landscape is located in epithelial cells, fibroblasts, and the surrounding extracellular matrix (ECM) of the female urogenital tract. Four main biological processes operate within the landscape, i.e., epithelial-mesenchymal transition (EMT), immune response activation, modulation of the ECM, and fibroblast survival and apoptosis. These processes interact with each other and are regulated through signaling cascades involving female sex hormones and the cytokine transforming growth factor beta 1 (TGFB1). In the legend of Figure 1, a succinct description of these four processes is given, and in the Supplementary Information, a detailed description of the molecular signaling cascades and protein interactions in the landscape is provided.

### 2.3. Enrichment Analyses of Differential Expression Data from an Independent POP Patient Cohort

The upstream regulator analysis of the 618 genes that were differentially expressed between POP and non-POP tissues in the independent cohort of 12 premenopausal women with POP (see Materials and Methods) at a corrected *p* < 0,01 (Supplementary Tables S2 and S3) yielded five regulators that are also present in the POP landscape: TGFB1, SMAD3, PGR and ESR2 are predicted to be activated while COL18A1 is predicted to be inhibited (Table 3). Moreover, the targets of TGFB1 are most significantly enriched within the set of 618 genes. We identified five functional gene categories that are present in the POP landscape and are enriched within the 618 differentially expressed genes: three categories relating to (epithelial) cell death—i.e., ‘apoptosis’, ‘necrosis of epithelial tissue’ and ‘cell death of epithelial cells’—are predicted to be inhibited, while two categories relating to (fibroblast) survival—i.e., ‘cell survival’ and ‘differentiation of fibroblasts’—are predicted to be activated (Table 4). We also identified five genes that encode proteins within the POP landscape and are differentially expressed between POP and non-POP tissues in the independent POP patient cohort, i.e., *AKR1C1, PLAUR*, and *VIM* are upregulated while *FBLN5* and *RAD52* are downregulated (Supplementary Table S4).

### 2.4. Metformin Downregulates the Expression of TGFB1 Target Genes in Vaginal Fibroblasts from POP Patients

**Figure 2:**
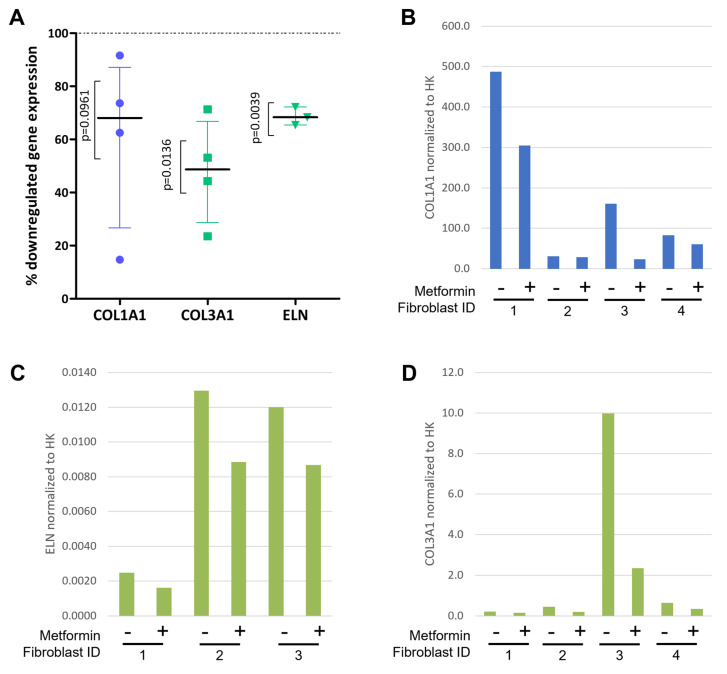
Metformin downregulates TGFB1-induced gene expression in fibroblasts from women with POP. Cells derived from prolapsed tissues from premenopausal women (*n* = 4) were stimulated for 24 h with TGFB1 [0.1 ng/mL] and then challenged with Metformin [2 mM] for another 24 h. Real-time PCR was used to measure three TGFB1-regulated genes from the landscape, i.e., the collagen genes *COL1A1* and *COL3A1* and *elastin (ELN)* and the two housekeeping genes *YWHAZ* and *HPRT1*. (**A**) shows the percentage (%) of downregulated gene expression in TGFB1-stimulated fibroblasts (with the dotted line corresponding to 100% gene expression in cells treated with TGFB1 [0.1 ng/mL]). One outlier was identified using Grubbs’ test [18] and removed from the ELN gene expression data set (n = 3). (**B**–**D**) show the expression of *COL1A1*, *ELN*, and *COL3A1* in the fibroblasts from each individual woman with POP normalized to the housekeeping genes according to the Pfaffl model [19], respectively.

As TGFB1 is the ‘master regulator’ of the POP landscape and its activation results in many landscape processes that are involved in POP, we tried to confirm metformin—an oral anti-diabetic drug that counteracts TGFB1—as a drug target ‘lead’ for POP by testing its effect on the expression of selected genes from the landscape. Since metformin suppresses TGFB1 responses specifically in fibroblasts cell lines [17], we used human vaginal fibroblasts from four independent POP patients to see if the drug could have the same beneficial effect in POP. As shown in Figure 2, we found that metformin (negatively) affects the TGFB1-induced expression of three ECM proteins in these fibroblasts: *COL3A1* (*p* = 0.0136) and *ELN* (*p* = 0.039) were significantly downregulated, while there was a tendency towards lower expression of *COL1A1* (*p* = 0.0961).

## 3. Discussion

We performed the first exome chip study of POP and integrated the results with the available genetic and expression data into a protein interaction landscape of the disease. This molecular landscape contains the proteins encoded by 26 of the 54 genes affected by SNVs from the exome chip study, as well as 41 other POP candidate molecules and two POP-implicated microRNAs. The POP landscape reveals four main biological processes that operate in epithelial cells, fibroblasts, and the surrounding ECM of the female urogenital tract: EMT, immune response activation, ECM modulation, and fibroblast survival and apoptosis. During pregnancy and (vaginal) delivery, similar changes in immune response activation and ECM modulation than in the POP landscape occur [20,21], which is interesting as increased parity and vaginal delivery are established environmental risk factors for developing POP [5].

Of note, none of the “classical” POP candidate genes—identified through candidate gene association studies and mainly encoding ECM proteins—or the genes from two previous GWASs of POP (including the landscape genes *COL18A1*, *FBLN3*, *NOP56*, *TBX5*, and *WNT4)* contained significant SNVs in our study.

Our landscape does not assume or represent a “sequence of events”, i.e., a number of biological processes and signaling cascades that occur in a spatially and temporally distinct order. Instead, a deficit in any or a combination of the four main landscape processes or their constituting signaling cascades, possibly aggravated through environmental risk factors and caused by variants in one or more genes, can lead to POP in various degrees of severity. This is reflected by the clinical reality of POP as a disease with a multifactorial etiology and variable treatment response.

Multiple signaling cascades in the landscape are regulated by female sex hormones (i.e., estrogen in its active form, estradiol, and progesterone). These hormones indirectly affect oxidative stress levels in epithelial cells and fibroblasts of the urogenital tract, as progesterone-activated PGR [22] and estradiol-activated ESR2 [23] upregulate the expression of the enzyme GGCT. Together with three other enzymes from the landscape—GPX1, GSTP1, and METAP1—GGCT is involved in the metabolism of glutathione [24], an important antioxidant that prevents oxidative stress-induced cellular damage by reactive oxygen species (ROS) [25]. Interestingly, ROS production by fibroblasts of women without POP was found to be increased through mechanical stress [26], while oxidative stress biomarkers were also increased in the uterosacral ligaments of POP patients [27]. ROS can also directly induce EMT [28], corroborating the role of oxidative stress in POP. Although sex hormones influence many landscape cascades, their role in POP etiology and, hence, their clinical relevance, is not completely understood. Increasing age and postmenopausal state are risk factors for POP [4,5], and the hypoestrogenic state after the menopause could be a factor in disease onset. Both lower serum levels of estradiol [29,30,31] and lower serum and urine levels of progesterone (metabolites) [29,32] have been associated with an increased risk of POP. Therefore, hormone replacement therapy (HRT) in postmenopausal women could have a favorable effect on disease progression or even prevent the disease from developing. However, studies on the effect of HRT in POP patients show conflicting results, as negative [33], protective [34], or no effects [35,36,37] have all been reported. Given the above, we suggest that future studies on the effect of HRT on POP take into consideration confounding factors such as endogenous sex hormone levels, menopause duration, or known associated genetic variations.

In addition to sex hormones and oxidative stress, the cytokine TGFB1 is an important landscape protein, as it represents the ‘master regulator’ that, when activated, affects and modulates all four biological processes in the landscape. Notably, (activated) TGFB1 is involved in ECM remodeling and regulating fibroblast function. Moreover, TGFB1 itself has an important role in POP etiology, and TGFB1 is both differentially expressed [31,38] and could have a functional effect—e.g., through affecting fibroblast proliferation [39]—in POP patients.

We have been able to corroborate our findings by analyzing differential gene expression data from an independent POP cohort. Our analysis of these data revealed that four ‘upstream regulators’—the most significant regulator TGFB1 and three other proteins that are important in EMT, i.e., SMAD3 and the sex hormone receptors PGR and ESR2—are predicted to be activated in POP tissue, while COL18A1, a collagen with multiple roles in ECM modulation, is predicted to be inhibited. These results are in line with the landscape, in which EMT and ECM modulation are two of the four main processes. Moreover, five genes encoding proteins in the landscape—including *AKR1C1* and *RAD52*, two genes from the exome chip study—are differentially expressed in the independent cohort, providing a direct validation of these genes as POP candidates. Further, our findings from the enrichment analysis of functional gene categories indicate that both epithelial cells and fibroblasts are affected in POP.

As the ‘master regulator’ of the landscape, TGFB1 would be an interesting target for developing novel treatments for POP. More specifically, drugs that lower TGFB1 activity could have beneficial effects and eventually be used in a clinical setting. However, as a result of its diverse effects on multiple signaling cascades in the body, drugs (negatively) targeting the expression or function of the genes encoding TGFB1 or its receptors are likely to be associated with multiple side effects. Hence, it would be better to test drugs that selectively target individual signaling pathways downstream of TGFB1. Such a novel treatment for POP may be metformin, an oral anti-diabetic drug that affects multiple genes/proteins, and processes in the landscape. First, metformin reduces the expression of TGFB1 [40,41] and decreases the TGFB1-promoted loss of CDH1 [42,43], which results in less EMT. Metformin also regulates female sex hormone signaling as it suppresses the expression of miR-221 [44,45] and miR-222 [44], two POP-implicated microRNAs that downregulate ESR1 expression. Metformin inhibits the TGFB1-induced synthesis of type 1 collagen by fibroblasts through suppressing SMAD3 phosphorylation [46,47,48] (a protein that has been found to be upregulated in POP tissues [49]). Furthermore, metformin may have a (more) directly beneficial effect on cells from the urogenital tract (and hence on POP) as it was found to reduce estradiol-induced EMT [50], inflammation [51], and age-associated dysfunction [52] of uterine cells. Therefore, since metformin also specifically suppresses TGFB1 responses in fibroblast cell lines [17], we used fibroblasts from patients to assess if the drug could affect TGFB1 signaling in a direction that might have a beneficial effect on POP (or POP development). We indeed showed that metformin counteracts the molecular mechanisms downstream of activated TGFB1 that negatively affect POP, through downregulating the expression of the key ECM (and landscape) proteins COL1A1, COL3A1, and ELN. These in vitro findings add evidential weight to metformin as a novel POP treatment, and further (pre)clinical studies are needed to fully develop it as a repurposed drug.

The current results should be viewed in light of some strengths and limitations. The main limitations of our study are that we have no independent replication of the exome chip results and we have only initial in vitro findings for the beneficial effect of one drug that could be repurposed for POP treatment. However, our findings are strengthened by the enrichment analyses of differential expression data from an independent POP cohort. In addition, our findings for metformin by no means show clinical effectiveness but could serve as the starting point for further studies on this and other drugs that could be repurposed.

## 4. Materials and Methods

### 4.1. Exome Chip Study

#### 4.1.1. Participant Characteristics and Quality Control

A case-control design was used for the exome chip study. Data on 242,901 genetic markers and 2478 individuals were available prior to the implementation of Quality Control (QC), which was implemented using PLINK software, version 1.9. Of the 2478 individuals, 548 were classified as cases, while the remaining 1930 individuals were considered to be controls. Given the nature of the studied phenotype, all cases were female, while the controls consisted of 977 females and 953 males. Prior to QC, the total genotype calling rate was 0.999053. A total of 12 individuals (11 cases and 1 control) were removed for a low per-individual calling rate (<0.95), while the same per-marker calling rate criterion (<0.95) led to the removal of 82 markers. Further QC steps in the combined case/control population included checks of markers for low minor allele frequency (MAF) (MAF < 0.005, failed by 199,851 markers), deviation from Hardy-Weinberg equilibrium (HWE) (*p* < 10^−4^, 280 markers), differential missingness with respect to phenotype (via--test-missing, *p* < 10^−3^, 88 markers), and haplotype (via--test-mishap, *p* < 10^−6^, 14 markers). Furthermore, individuals were checked for sex discordance (via--check-sex, 10 individuals), individual duplicity or high-relatedness (via--genome, PI_HAT ≥ 0.9 [53], 7 individuals), and heterozygosity (via--het, with conf. int. based on MAD, 13 individuals). Heterozygous haploid genotypes and non-missing non-male Y chromosome genotypes were set to missing values. Lastly, all markers that are not located within a gene were removed (8732 markers). After all the QC steps and removing all the male controls—to allow for a ‘clean’ comparison with the all-female POP cases—there were 33,854 markers and 1486 females (526 cases and 960 controls) left for the exome chip analysis. The case group of 526 women presented with symptoms and signs of POP (including women with recurrent POP and previous POP surgery) at the Department of Obstetrics and Gynecology of the Radboud University Medical Center (Radboudumc), Nijmegen, The Netherlands, in January 2007–February 2011. Signs of POP were defined as stage II or more, according to the Pelvic Organ Prolapse Quantification (POP-Q) system [54], i.e., the descent of the leading edge of the prolapse 1 cm above the hymenal remnants or beyond. POP had already been surgically treated in some women at the time of inclusion. Exclusion criteria were a poor understanding of the Dutch language and/or genetic diseases with an increased risk of POP (e.g., Ehlers-Danlos and Marfan syndromes, and myotonic dystrophy type 1). For POP cases, we collected data on previous surgery, POP-Q stage, as well as POP complaints measured with the validated Dutch translation of the Urogenital Distress Inventory (UDI) [55]. If more than one measurement was available over time, the most severe measurement was used. The UDI records the presence or absence of specific symptoms, and associated bother on a four-point Likert scale. The UDI scores are transformed to a continuous scale, ranging from 0 (symptom absent or no bother) to 100 (a very bothersome symptom). According to this scale, women without symptoms and women with symptoms but without any bother are scored equally at 0. The exome chip study was approved by the local institutional review board in an amendment of CMO numbers 2007/043 and 2010/071. As the control group, we used the exome chip data available from 960 women from the Nijmegen Biomedical Study (NBS), a general population-based survey conducted by Radboudumc’s Departments for Health Evidence and Department of Laboratory Medicine [56]. For the cases and controls, data on age, parity, and BMI were collected.

#### 4.1.2. Laboratory Analysis and Genotyping

Blood samples were drawn from all cases and controls. Genomic DNA was isolated from peripheral blood using the Nucleospin Blood Kit (Macherey-Nagel^®^, Düren, Germany). Both case and control samples were genotyped (Illumina Exome BeadChip v1.1, Illumina^®^ San Diego, CA, USA) and called at the Human Genome Facility and the department of Epidemiology, Erasmus MC, Rotterdam. Calling was performed in GenomeStudio using the default cluster files provided by Illumina (HumanExome-12v1-1_A.egt), followed by the calling of non-called variants using zCall [57].

#### 4.1.3. Population Structure

Since principal component analysis uncovered evidence of population structure in the data, we performed clustering analysis using the R function hclust() with method ward.D [58]. The dissimilarity structure was generated by dist() using the first 5 components produced by PLINK’s multidimensional scaling (MDS) analysis (via--mds-plot 10) to estimate continuous axes of ancestry. Using the output of the R function NbClust(), the desired number of clusters was set to 3. The case/control counts in the resulting three clusters were 96/182,124/195 and 306/583.

#### 4.1.4. Statistical Analysis

Using the quality controlled clustered data, we analyzed the 33,854 markers for association with POP using the Cochran-Mantel-Haenszel (CMH) test’s asymptotic *p*-values via PLINK’s --mh option at the Bonferroni-corrected level of significance (0.05/33,854 = 1.48 × 10^−6^).

### 4.2. Molecular Landscape Building

Applying the approach that we previously used for multiple neurodevelopmental and neurological disorders [59,60,61], we then built a molecular landscape of POP. First, we searched the literature for the (putative) function of all candidate genes implicated through the exome chip study as well as other POP candidates implicated via other evidence, including (genome-wide) genetic association studies, miRNA/mRNA/protein expression studies, and/or genetic animal model studies (Supplementary Table S1). For this literature analysis, we used the UniProt Protein Knowledge Base (http://www.uniprot.org/uniprot accessed on 21 June 2022) to gather basic information on the function of all candidate genes and their encoded proteins. Based on the biological processes that have already been suggested to be impaired in POP, we then searched PubMed (http://www.ncbi.nlm.nih.gov/sites/entrez accessed on 21 June 2022) for all genes/proteins and using the search terms “collagen”, “connective tissue”, “fibrosis”, “pelvic” and “pelvic organ”. Further, we searched PubMed for all functional interactions among all candidate proteins. Lastly, based on all the gathered information, we generated a molecular landscape of protein interactions. The figure of the landscape was made using the drawing program Serif DrawPlus version 4.0 (www.serif.com accessed on 21 June 2022).

### 4.3. Enrichment Analyses of Differential Gene Expression Data from an Independent POP Patient Cohort

In order to corroborate our findings, we conducted enrichment analyses of genes that were differentially expressed between POP and non-POP tissues in POP patients. For this, we used microarray data that had been previously deposited by authors MHK and AMRZ on the GEO website (http://www.ncbi.nlm.nih.gov/gov/geo/ accessed on 21 September 2022). The data set GSE53868 contains microarray-derived gene expression data from 12 premenopausal women with POP, and the data are available for POP (prolapsed tissue) and non-POP (peri-cervical region) tissues from the anterior vaginal wall of each patient. To identify expression changes between POP and non-POP tissues, we performed differential gene expression analysis using R statistical software version 4.0 and the R/Bioconductor [62] package limma [63]. Since there were two arrays per individual patient, we used the “paired samples” design, allowing for correlation analyses within patients. The Benjamini-Hochberg (BH) method was used to correct for multiple testing, and only protein-coding genes with an adjusted *p*-value <0.01, independent of magnitude of change, were considered as differentially expressed in pair-wise comparisons between POP and non-POP sites, and were used in the subsequent enrichment analyses.

First, we used the Ingenuity Pathway Analysis (IPA) software (winter release 2022) (Qiagen Bioinformatics, https://www.qiagenbioinformatics.com/products/ingenuity-pathway-analysis/ accessed on 21 December 2022) to perform gene enrichment analyses of the 618 unique genes that were differentially expressed between the POP and non-POP tissues of 12 POP patients at a BH-corrected *p*-value < 0.01 (Supplementary Table S2). We then conducted an upstream regulator analysis to identify the proteins that are both present in the POP landscape and regulate the expression of multiple targets within the set of 618 genes. Based on the ‘‘Ingenuity Knowledge Base’’, a repository of data from publicly accessible databases and data that are manually curated by systematically reviewing published literature, IPA can generate a list of ‘upstream regulators’, i.e., proteins or compounds that regulate the expression of multiple target genes, from an input list. The program also calculates a z-score based on the expression changes of the input genes, which is a measure of the directionality of the upstream regulator. In this respect, a z-score ≥2.00 and ≤−2.00 (reflecting activation and inhibition of the upstream regulator-dependent effects on target gene expression, respectively) is considered to be significant. IPA also calculates a *p*-Value that reflects how enriched the targets of each upstream regulator are in the differentially expressed genes. In addition to generating a list of upstream regulators, IPA assigns genes and their corresponding proteins to functional categories and, as with the upstream regulators, we identified those functional categories that are both present in the POP landscape and are enriched in the set of 618 genes. IPA also generated z-scores—reflecting whether the functional gene category was activated or inhibited—as well as BH-corrected P-values of enrichment for each category. Further, for all genes in the POP landscape, we searched the list of all differentially expressed genes (Supplementary Table S3) to ascertain whether their expression was (significantly) upregulated or downregulated (Supplementary Table S4).

### 4.4. In Vitro Experiments and Statistical Analysis

In this study, we used primary human vaginal fibroblasts (passages 6–7) from the prolapsed anterior vaginal wall of four premenopausal women with cystocele that were previously isolated from our group [64]. Full thickness biopsies (>1 cm^2^) from the anterior vaginal wall were retrieved from each of the women. Biopsies were collected in PBS at 4 °C, and within 24 h, primary human vaginal fibroblasts were isolated, as previously described [65]. Experiments were performed in an incubator at 37 °C, 95% humidity, and 5% CO_2_. A cell density of 10,000 cells/cm^2^ were seeded on 6-well plates and cultured for 24 h with the cell culture medium DMEM (Gibco-Life technologies, Paisley, UK) supplemented with 10% fetal bovine serum (FBS; Sigma-Aldrich, St. Louis, MO, USA), 100 μg/mL streptomycin, 100 U/mL penicillin, and 250 μg/mL amphotericin-B (Sigma). Subsequently, the media was refreshed with cell culture media supplemented with 0.1 ng/mL of human TGF beta 1 recombinant protein (eBioscience, Thermo Fisher Scientific, Waltham, CA, USA). After 24 h of culture, cells were stimulated (or not) with 2 mM of metformin hydrochloride (Tocris Bioscience, Bristol, UK) for an additional 24 h. At the end of the experiment, total RNA was isolated using TRIzol and following the manufacturer’s instructions (Invitrogen). Concentration and purity of the RNA were determined with a Nanodrop-1000 spectrophotometer (Thermo Scientific, Waltham, CA, USA). Subsequently, 2 ug DNase-I-treated total RNA was used to make cDNA using random hexamer primers (Roche) and SuperScript II Reverse Transcriptase (Invitrogen). Real-time PCR (RT-PCR) was performed with a LightCycler 480 SYBR Green I master mix and in a LightCycler LC480 device (Roche) with the following amplification conditions: 5 min at 95 °C, followed by 45 cycles of 10 sec at 95 °C, 20 sec at 60 °C, and 20 sec at 72 °C. Crossing-point (Cp) values were determined using the LightCycler480 SW, version 1.5 software (Roche). The gene expression levels for three selected genes from the landscape—the collagen genes *COL1A1* and *COL3A1*, and *elastin* (*ELN*)—were normalized to the housekeeping genes *YWHAZ* and *HPRT1* and were calculated for the vaginal fibroblasts from each of the women with POP separately and according to the mathematical model for relative quantification in RT-PCR described by Pfaffl [19]. *COL1A1*, *COL3A1* and *ELN* were selected as they encode key, TGFB1-induced components of the ECM surrounding fibroblasts in the anterior vaginal wall that regulate the mechanical strength of this wall, and as such, are dysregulated in POP. Further, to find the right housekeeping genes, we first performed a pilot experiment in which we tested which common housekeeping genes show stable expression in vaginal fibroblasts regardless of the conditions these cells are in [66]. *YWHAZ* and *HPRT1* turned out to be the most stable and were therefore chosen as reference housekeeping genes. The primers for the three landscape genes and the two housekeeping genes were acquired from Life Technologies and are listed in Supplementary Table S5. The results were calculated as the percentage of downregulated gene expression by metformin in TGFB1-stimulated fibroblasts and are presented in a scatter plot. A one-sample *t*-test compared to 100 was used to test the effect of metformin on TGFβ-stimulated cells with the software Prism version 5.03 (GraphPad Software Inc., San Diego, CA, USA). We also applied Grubbs’ test [18] for outlier correction.

## 5. Conclusions

Based on the results of our exome chip study and available literature data, we built a molecular landscape of POP that provides novel insights into the biological processes underlying the disease and that we could corroborate through analyzing gene expression data from an independent cohort. In addition, the POP landscape provides interesting leads for existing drugs such as metformin that could be further developed as repurposed compounds in selected patient groups.

## Figures and Tables

**Table 1:** Characteristics of POP cases and controls that were analyzed in the exome chip study.

Characteristic	Cases (*n* = 526) *	Controls (*n* = 960) *	*p*-Value
Age ^a^ (years)	51.3 ± 13.5 [21–87; 522]	56.2 ± 10.8 [27–75; 960]	<0.01 ^e^
Parity	2.4 ± 1.1 [0–9; 515]	2.3 ± 1.1 [0–10; 791]	0.47 ^e^
BMI (kg/m^2)^	25.6 ± 3.9 [15–42; 393]	25.4 ± 4.3 [17.6–50.1; 937]	0.29 ^e^
Previous POP surgery		unknown	NA
0	277 (52.8; 525)		
1	150 (28.6; 525)		
≥2	98 (18.7; 525)		
POP-related complaints ^b^	48.9 ± 32.3 [0–100; 323]	unknown	NA
POP-Q stage ^c^		unknown	NA
Stage 0	2 (0.4; 502)		
Stage I	12 (2.4; 502)		
Stage II	217 (43.2; 502)		
Stage III	248 (49.4; 502)		
Stage IV	23 (4.6; 502)		
Postmenopausal ^d^	278 (73; 380)	771 (81; 955)	<0.01 ^f^

* Data are presented as mean ± standard deviation [range; n] in the case of continuously distributed data and as absolute numbers (percentages; n) in the case of categorical data. ^a^ Age at diagnosis for cases, age at time of survey/sampling for controls. ^b^ Measured with and reported as ‘total score’ from the validated Dutch translation of the Urogenital Distress Inventory (UDI). In cases of repetitive measurements over time, the worst UDI score available in the medical file was used. ^c^ Because the genetic background does not change over time, women were included in the exome chip study as a POP case at any time after diagnosis. This could also be after successful reconstructive surgery, which explains the lower stages in some POP cases (who had already been surgically treated). In all POP cases, the worst stage available in the medical file was used, and this information was available for 502/526 cases. ^d^ This was assessed through the NBS participants’ answer to the question: ‘Did you ever experience menopausal symptoms (e.g., hot flushes)?’, with possible ‘yes’, ‘no’, or ‘not applicable’ (I am not in the menopause yet). ^e^ *p* value is calculated with an independent samples *t*-test. ^f^ *p* value calculated with the chi-square test. Abbreviations: BMI, body mass index; NA, not applicable; POP, pelvic organ prolapse; POP-Q, pelvic organ prolapse quantification system.

**Table 2:** Top 54 SNVs identified through our exome chip study. For each SNV, the minor allele frequencies in the POP cases and controls (MAF-ca and MAF-co), the *p* value of association with POP, and the Bonferroni-corrected *p* value are provided. All SNPs with Bonferroni-corrected *p* < 0.05 are listed. In addition, the affected gene, SNV type, nucleotide change, and amino acid change are indicated. If applicable and available, the Grantham and CADD scores—which are both predictive measures of pathogenicity—for each SNV are given. Further, the 26 genes encoding proteins within the molecular landscape of POP (Figure 1) are indicated in bold.

SNV	MAF-ca ^a^	MAF-co ^a^	*p*	Corr *p*	Gene	Type	Nucleotide Change	Amino Acid Change	GS ^b^	CADD ^c^
rs138799629	0	0.13650	6.25 × 10^−36^	2.11 × 10^−31^	*ANKRD49* ^d^	missense	G > A	p.Val46Ile.	29	14.58
rs115986826	0.08175	0.00208	7.41 × 10^−34^	2.51 × 10^−29^	***NEB***	missense	T > G	p.Asp1329Ala	126	24.70
rs138533962	0.00380	0.13490	2.96 × 10^−33^	1.00 × 10^−28^	***STYK1***	missense	G > A	p.Arg379Cys	180	25.2
rs149745899	0.00285	0.11880	2.17 × 10^−29^	7.35 × 10^−25^	***SCUBE2***	missense	G > A	p.Ser481Phe.	155	18.81
rs140794579	0.03422	0.17660	9.56 × 10^−29^	3.24 × 10^−24^	***ACN9***	missense	A > C	p.Gln97Pro	76	26.4
rs199737061	0.01616	0.13700	5.54 × 10^−27^	1.88 × 10^−22^	*ZNF700* ^e^	missense	A > C	p.His685/703Pro	77	0.001
rs138091786	0.00856	0.12140	8.10 × 10^−27^	2.74 × 10^−22^	*LRRC34*	missense	A > G	p.Val308/324/337/369Ala.	64	14.38
rs114675387	0.00382	0.10890	4.07 × 10^−26^	1.38 × 10^−21^	*MUC17*	missense	A > G	p.Thr2611Ala	58	0.002
rs61748812	0.05133	0.17760	3.62 × 10^−22^	1.22 × 10^−17^	***NACA2***	missense	C > A	p.Val9Phe	50	17
rs150552771	0	0.07865	1.25 × 10^−20^	4.25 × 10^−16^	*LPGAT1* ^f^	missense	T > C	p.Lys200Glu.	56	19.09
rs151097728	0.00095	0.08021	1.96 × 10^−20^	6.64 × 10^−16^	***TFAM***	missense	C > G	p.Gln82/100Glu	29	0.013
rs11828907	0.04373	0.00052	1.34 × 10^−19^	4.54 × 10^−15^	***AHNAK***	missense	T > C	p.Asp4304Gly	94	22.60
rs144392720	0.04183	0	1.51 × 10^−19^	5.13 × 10^−15^	***TNFRSF10C***	missense	A > G	p.Gln58Arg	43	3.575
rs200940950	0.00190	0.07500	1.61 × 10^−18^	5.46 × 10^−14^	***METAP1***	splice site	C > T	-	NA	5.733
rs79324787	0	0.06719	9.80 × 10^−18^	3.32 × 10^−13^	*KIF27*	missense	T > C	p.Ile890/921/987Val	29	2.241
rs142757633	0	0.06719	1.05 × 10^−17^	3.57 × 10^−13^	***YWHAB***	missense	A > C	p.Lys77Gln	53	17.12
rs78707986	0.00190	0.07031	2.63 × 10^−17^	8.91 × 10^−13^	*GXYLT1*	missense	A > C	p.His117/148Gln	24	22.30
rs141705162	0.00095	0.06667	6.02 × 10^−17^	2.04 × 10^−12^	***FASTKD1***	missense	G > A	p.Ala320/343Val	64	19.38
rs76146040	0.00570	0.07604	1.17 × 10^−16^	3.96 × 10^−12^	*OR5H14*	missense	G > A	p.Met234Ile	10	0.051
rs34429135	0.03707	0.13120	1.58 × 10^−16^	5.34 × 10^−12^	***LAIR2***	missense	T > A	p.Phe115Tyr.	22	0.001
rs142631876	0	0.06094	4.05 × 10^−16^	1.37 × 10^−11^	***RRM2***	missense	A > G	p.Arg342/402Gly	125	19.83
rs145531717	0.00095	0.06302	4.30 × 10^−16^	1.45 × 10^−11^	***ATP12A***	missense	C > A	p.Arg490/496Ser	110	23.80
rs151149890	0	0.05885	1.10 × 10^−15^	3.71 × 10^−11^	*LRRC37A3*	missense	G > A	p.Thr570/631/711/1593Met	81	0.003
rs200909708	0.00570	0.07240	1.12 × 10^−15^	3.79 × 10^−11^	***AKR1C1*** ^g^	missense	G > A	p.Val118/151/153Met	21	0.133
rs146515657	0.05038	0.00573	1.72 × 10^−15^	5.83 × 10^−11^	***USP4***	missense	T > C	p.Asn389/603/650Ser	46	3.361
rs190805303	0.36410	0.50780	5.08 × 10^−14^	1.72 × 10^−9^	*CCDC90B*	missense	T > C	p.Thr90/118/182/191Ala	58	0.131
rs144738877	0	0.05312	3.07 × 10^−14^	1.04 × 10^−9^	*IFT80*	missense	C > T	p.Arg44/116/253/424His	29	27.90
rs4362173	0.03517	0.11090	1.16 × 10^−12^	3.92 × 10^−8^	*OR52E6*	missense	T > C	p.Ile39/43Val	29	0.001
rs149907018	0	0.04740	7.55 × 10^−13^	2.55 × 10^−8^	*SCN4A*	missense	T > C	p.Ile430Val	29	5.153
rs138362833	0.40210	0.53650	2.93 × 10^−12^	9.91 × 10^−8^	*PRSS1*	missense	A > C	p.Lys237/251Asn	94	9.898
rs114501427	0	0.04531	2.77 × 10^−12^	9.36 × 10^−8^	***SPOPL***	missense	G > A	p.Asp349Asn	23	20.90
rs2302212	0.05989	0.13330	6.23 × 10^−10^	2.11 × 10^−5^	*NCAPG*	missense	T > C	p.Met231Thr	81	15
rs144426665	0	0.03750	2.70 × 10^−10^	9.15 × 10^−6^	*OR5B17*	missense	C > A	p.Val124Leu	32	25.50
rs115910467	0.00286	0.04271	5.77 × 10^−10^	1.95 × 10^−5^	***GGCT***	missense	C > T	p.Arg108/160His	29	17.43
rs79820636	0.40820	0.52190	2.94 × 10^−9^	9.96 × 10^−5^	*PCMTD1*	missense	C > T	p.Arg172/248His	29	34
rs4987208	0.04485	0.01042	1.53 × 10^−9^	5.18 × 10^−5^	***RAD52*** ^h^	stop-gain	A > C	p.Tyr415Ter	NA	35
rs201483250	0.00761	0.05052	2.04 × 10^−9^	6.90 × 10^−5^	***LILRA1***	missense	C > T	p.Arg179/196Trp	101	10.48
rs201702426	0.01616	0.06458	2.43 × 10^−9^	8.23 × 10^−5^	***AKR1D1***	missense	T > C	p.Ile63/119Thr	89	25.90
rs9871143	0.56840	0.46090	2.14 × 10^−8^	7.23 × 10^−4^	*OR5H6*	missense	G > A	p.Asp285Asn	23	12
rs4252119	0	0.03281	3.28 × 10^−9^	1.11 × 10^−4^	***PLG***	missense	C > T	p.Arg408Trp	101	0.880
rs200020201	0.00095	0.03490	3.33 × 10^−9^	1.13 × 10^−4^	*NKD1*	missense	A > G	p.Met130Val	21	0.089
rs200454083	0.02376	0.07708	3.63 × 10^−9^	1.23 × 10^−4^	***ANKLE2***	missense	G > C	p.Pro175/758/820Ala	27	0.001
rs17115182	0	0.03125	7.78 × 10^−9^	2.63 × 10^−4^	*KRR1*	missense	G > A	p.Pro43Ser	74	24.40
rs201566504	0	0.03021	1.75 × 10^−8^	5.91 × 10^−4^	*ZFP14* ^i^	missense	C > T	p.Val322Ile	29	23.00
rs11229158	0.00095	0.02552	5.43 × 10^−7^	1.84 × 10^−2^	*OR4A16*	missense	C > A	p.Leu188Ile	5	8
rs141135045	0	0.02865	3.67 × 10^−8^	1.24 × 10^−3^	*RFESD*	missense	C > G	p.Pro81/134Arg	103	31.00
rs117389731	0.00951	0.04740	5.93 × 10^−8^	2.01 × 10^−3^	*ZNF780B*	missense	G > A	p.Thr542/690Ile	89	0.086
rs1051949	0	0.02760	6.81 × 10^−8^	2.31 × 10^−3^	***MRPL35***	missense	G > A	p.Arg29His	29	24.00
rs200482299	0.00095	0.02917	9.59 × 10^−8^	3.25 × 10^−3^	***ARHGEF19***	missense	C > T	p.Ala157/163/474Thr	58	24.20
rs200171449	0	0.02500	2.48 × 10^−7^	8.40 × 10^−3^	*TAS2R46*	missense	T > G	p.Ser170Arg	110	0.629
rs188761759	0.00856	0.04115	6.81 × 10^−7^	2.30 × 10^−2^	***EMILIN1***	missense	G > T	p.Ala391Ser	99	0.405
rs74951519	0.36410	0.50780	5.08 × 10^−14^	1.72 × 10^−9^	*OR7C2*	missense	A > G	p.Lys294Arg	26	0.002
rs144462785	0.00095	0.02552	6.93 × 10^−7^	2.34 × 10^−2^	*PCDHB12*	missense	T > G	p.Leu252Arg	102	0.036
rs17213351	0.00951	0.04219	7.62 × 10^−7^	2.58 × 10^−2^	*HLA-DQA2*	missense	G > A	p.Ala222Thr	58	22.40

Abbreviations: NA, not applicable; POP, pelvic organ prolapse; SNV(s), single nucleotide variant(s). ^a^ A MAF of ‘0’ indicates that none of the POP cases or controls were found to carry the minor allele of the SNV; ^b^ the Grantham score (GS) for missense mutation-type SNVs ranges from 0 to 215 and a higher score indicates a stronger negative effect of the amino acid change on protein structure and function [15]; ^c^ the combined annotation-dependent depletion (CADD) tool combines 63 distinct computational prediction methods—including the GS for missense mutations—and calculates a single pathogenicity score [14]. SNVs with CADD scores > 20 and > 10 indicate that these are predicted to be among the 1 and 10% most deleterious of all possible substitutions in the human genome, respectively [14,16]. Within our set of differentially expressed genes in an independent POP cohort (see Methods), the expression of six of the genes from the exome chip was different: the expression of *ANKRD49* was downregulated (FC = −1.19; corrected *p* = 7.08 × 10^−3^) ^d^, the expression of *ZNF700* was downregulated (FC = −1.13; corrected *p* = 2.27 ×−02) ^e^, the expression of *LPGAT1* was upregulated (FC = 1.19; corrected *p* = 1.70 × 10^−2^) ^f^, *AK1RC1* was upregulated (FC = 1.55; corrected *p* = 7.01 × 10^−3^) ^g^, the expression of *RAD52* was downregulated (FC = −1.16; corrected *p* = 3.77 × 10^−2^) ^h^, and the expression of *ZFP14* was downregulated (FC = −1.32; corrected *p* = 3.94 × 10 ^−2^) ^i^.

**Table 3:** Upstream regulator analysis of the 618 mRNAs that were differentially expressed (corrected *p* < 0.01) between POP and non-POP tissues in 12 POP patients (see Materials and Methods). The 5 upstream regulators that are present in the POP landscape are listed with a z-score ≥ 2.00 or ≤ −2.00, indicating that they are predicted to be activated or inhibited, respectively. For each regulator, the *p*-value—referring to how enriched the targets of each upstream regulator are in the differentially expressed mRNAs—and the number of target genes are also listed.

Upstream Regulator	z-Score	P	Number of Target Genes
TGFB1	2.92	1.16 × 10^−9^	11
SMAD3	3.67	2.03 × 10^−9^	10
PGR	2.84	5.63 × 10^−6^	10
ESR2	2.02	6.90 × 10^−3^	10
COL18A1	−2.41	3.30 × 10^−2^	6

**Table 4:** Functional gene categories that are present in the POP landscape and enriched within the 618 mRNAs that were differentially expressed (corrected *p* < 0,01) between POP and non-POP tissues in 12 POP patients (see Section 4). The 5 categories are listed with a z-score ≥2.00 or ≤−2.00, indicating that they are predicted to be activated or inhibited, respectively. For each category, the BH-corrected *p*-value of enrichment and the number of genes in the category are also listed.

Gene Category	z-Score	BH-Corrected P	Number of Genes
Apoptosis ^a^	−2.47	1.23 × 10^−12^	185
Cell survival ^a^	2.34	2.06 × 10^−7^	108
Necrosis of epithelial tissue ^a^	−2.52	7.92 × 10^−5^	49
Differentiation of fibroblasts ^b^	2.55	7.63 × 10^−4^	11
Cell death of epithelial cells ^a^	−2.51	2.01 × 10^−3^	38

Abbreviations: POP, pelvic organ prolapse; BH, Benjamini-Hochberg. **^a^** This gene category falls under the broader ‘cell death and survival’ category. **^b^** This gene category falls under the broader ‘connective system development and function’ category.

## Data Availability

All the key data that support the findings of this study are available in the main text or the Supplementary Materials. Other, more extensive data are available from the corresponding author upon reasonable request.

## Supplementary Materials

The supporting information can be downloaded at: https://www.mdpi.com/article/10.3390/ijms24076087/s1.
