# Supplementary material for: Molecular Landscape of Pelvic Organ Prolapse Provides Insights into Disease Etiology
Source: Int J Mol Sci. 2023 Mar 23;24(7):6087. doi: 10.3390/ijms24076087 (PMC10094264; doi:10.3390/ijms24076087)
Supplement: Supplementary file 1 [file ijms-24-06087-s001.zip › ijms-2158288-supplementary/Supplementary Information.pdf]

Supplementary Information

Full description of POP landscape depicted in Figure 1

In the description of the landscape below, all candidate genes/proteins that were implicated in POP through our exome chip study (**Table 2**), the other POP candidate genes/proteins/molecules (**Supplementary Table 1**), and three POP-linked signaling molecules (estradiol, glutathione and progesterone) are indicated in bold.

Signaling through the molecular landscape is initiated at the cell membrane of epithelial cells and underlying fibroblasts of the urogenital tract, where the binding of ligands from the extracellular matrix (ECM) to their respective receptors leads to the modulation of several downstream molecular cascades in the cytoplasm, cytoskeleton, endoplasmic reticulum and nucleus. These cascades converge on regulating epithelial-mesenchymal transition (EMT). In essence, EMT is characterized by a so-called 'cadherin switch' that is initiated when epithelial cells start producing less of the epithelial marker and membrane protein **E-cadherin (CDH1)** and more of the mesenchymal marker N-cadherin (CDH2) (not shown)[1]. This in turn results in the adherens junctions between epithelial cells letting loose and the epithelial cells gradually transforming into mesenchymal cells.

In normally functioning epithelial cells, cytoplasmic **AHNAK** binds and stabilizes the epithelial cytoskeleton, which in turn results in **CDH1** being anchored to and modulated by the (actin) cytoskeleton. Cytoskeleton-anchored **CDH1** of opposite cells bind each other and hence positively regulate epithelial cell-cell contacts, preventing EMT[2,3]. Further, **NEB** binds and stabilizes the (actin) cytoskeleton[4], the organization of which is also regulated by cytoplasmic **ARHGEF19**[5], which is itself upregulated by the extracellular cytokine **TGF-beta-1** (**TGFB1**)[6]. **TGFB1** is a key positive regulator of EMT as it significantly downregulates **CDH1** expression[7]. In this respect, **SCUBE2** is an ECM protein that is involved in upregulating the expression of **CDH1**, and hence inhibits **TGFB1**-induced EMT[8]. Furthermore, **AHNAK** shuttles between the cytoplasm and nucleus[9], and nuclear **AHNAK** can *induce* EMT[2]. A possible explanation for the role of **AHNAK** in inducing EMT may lie in the fact that in response to **TGFB1**, **AHNAK** promotes the activation and translocation of **SMAD3** to the nucleus[10], which in turn downregulates **CDH1**[7].

In addition and as further described below, **SMAD3** regulates the expression of a number of EMT-involved extracellular proteins. ZFYVE9 (other name: SARA) is a peptidase that binds and retains **SMAD3** in the cytoplasm. Upon phosphorylation and activation by **TGFB1** (see above), phosphorylated **SMAD3** translocate to the nucleus and promotes EMT[11,12]. Further, ZFYVE9 binds and forms a functional complex with the endoplasmic reticulum (ER) membrane-located **ANKLE2**[13], and inhibits EMT through upregulating the expression of **CDH1**[14]. **TGFB1** is additionally involved in modulating the **SMAD3**-ZFYVE9-**ANKLE2** complex through downregulating the expression of ZFYVE9[14] and regulating **ANKLE2** expression[15].

Within epithelial cells, the activation and translocation of **SMAD3** to the nucleus is also positively regulated by the adaptor protein NFKB[16]. In turn, NFKB is activated by cytoplasmic **FASTKD1** and **RRM2**[17,18], and downstream of **TGFB1** signalling[19]. Furthermore, **SMAD3** and **TGFB1** upregulate each other's expression[20,21]. When activated in the nucleus by e.g. **AHNAK** (see above), **SMAD3** downregulates the expression of the nuclear estrogen receptor **ESR1**[22]. In turn, through binding and forming a complex with its natural ligand, the female sex hormone **estradiol (E)**, **ESR1** upregulates the expression of **AHNAK**[23,24]. The POP-implicated microRNAs **miR-221** and **miR-222** also negatively regulate **ESR1** expression through binding and destabilizing the **ESR1** mRNA[25]. Epithelial[26] **NOP56** also binds and interacts with estradiol-bound **ESR1**[24]. Moreover, **USP4** is an enzyme that deubiquitinates ADORA2A - an epithelial and mesenchymal receptor that is bound and activated by extracellular adenosine (A)[27] - which increases the amount of functional receptors at the cell membrane[28]. The A-ADORA2A complex is also involved in upregulating the expression of **CDH1**[29] and hence inhibiting EMT[30] (see above). Further, **STYK1**, a membrane protein that is upregulated downstream of **SMAD3**[31], promotes EMT through decreasing **CDH1** expression[32].

Apart from downregulating **ESR1** and **CDH1** (see above), nuclear **SMAD3** upregulates the expression of the other nuclear estrogen receptor **ESR2**[22]. Further, **E**-bound **ESR1** and **ESR2** upregulate and downregulate the expression of **PGR** - the nuclear receptor for the pregnancy hormone **progesterone (P)** - respectively[33]. Interestingly, through our exome chip analysis, we identified POP patients with mutations in the genes encoding **AKR1C1** and **AKR1D1**, two functionally related enzymes that negatively regulate the production of **P** through promoting its conversion into inactive **P** metabolites[27]. **P**-activated **PGR**[34] and **E**-activated **ESR2**[35]

upregulate the expression of **GGCT**, one of the major enzymes involved in the metabolism of the important cellular antioxidant **glutathione (G)**[36] that is highly expressed in the nucleus and cytoplasm of epithelial cells of the reproductive system[37]. **GPX1**, **GSTP1** and **METAP1** are other cytoplasmic enzymes with an important role in the production and metabolism of glutathione[27,38]. The membrane protein **RAGE** is the receptor for extracellular **AGE** (or advanced glycation end product) molecules. The **AGE**-**RAGE** complex regulates a number of downstream signaling cascades and is found in both epithelial cells and fibroblasts (see below)[39]. In epithelial cells, **GPX1** is involved in downregulating the expression of **RAGE**[40].

In addition, **GSTP1** is involved in regulating the expression of **ATP12A**[41], a membrane-located symporter of potassium (K+) and hydrogen (H+)[27] that regulates epithelial cell function[42]. Moreover, **E**-bound **ESR1**[43] and **ESR2**[44] as well as **P**-bound **PGR**[45,46] inhibit EMT through upregulating **CDH1** expression, while **extracellular matrix protein 1 (ECM1)** promotes EMT through downregulating **CDH1**[47]. **E**-bound **ESR1** also upregulates the expression of **WNT4**[48], an extracellular regulator of EMT[49] that is downregulated by **TGFB1**[6]. Further, **FBLN3** is an ECM protein that binds and forms a functional complex with **ECM1**[50] and negatively regulates EMT through upregulating **CDH1** expression[51]. In addition, **FBLN3** inhibits **TGFB1**-dependent signaling[52]. Lastly, **SDHAF3** is a mitochondrial enzyme that negatively regulates EMT[53]. In addition, **TFAM** is a mitochondrial enzyme that is highly expressed in epithelial cells[54] and required for maintaining normal levels of mitochondrial DNA (mtDNA)[27]. Interestingly, changes in mtDNA copy number may play a role in POP development[55-57].

A part of the landscape is also involved in regulating the immune response in that beta-2-microglobulin (B2M) - an EMT-inducing[58,59] extracellular protein that mediates the presentation of antigens to the immune system [27]- is (up)regulated by **TGFB1**[60]. Further, B2M binds and functionally interacts with **LILRA1**[61], and it is involved in upregulating the expression of **HLA-DQA1** and **HLA-DQB1**[62], which form a functional complex with each other[63] and are both downregulated by **TGFB1**[62]. **HLA-DQA1** and **HLA-DQB1** are MHC class II (MHC II) proteins[27] . MHC II proteins are expressed in epithelial cells of the female reproductive tract where they are involved in regulating the immune response through presenting foreign antigens to circulating T lymphocytes[64,65] (not shown).

Remodeling of the ECM constitutes another major signaling cascade within the landscape and almost all POP candidate genes identified prior to this study encode proteins that are involved in this process. Further, fibroblasts produce most of these extracellular POP candidates (see below). In addition, literature suggests that in women with and without POP, mechanical stretch alters the response of fibroblasts to ECM remodeling and the interaction between the ECM and these fibroblasts[66-68]. The ECM is composed of different molecules, including **collagen (COL)** and **elastin (ELN)** fibers as well as proteins that crosslink or regulate them. First, A-activated ADORA2A (see above) is involved in upregulating the expression of the extracellular collagen **COL3A1**[69,70] and downregulating the extracellular matrix metalloproteinase **MMP9**[71] - which itself is upregulated downstream of NFKB[72,73] and degrades **COL1A1**[74] and **ELN** [75] - and **TGFB1**[30]. Moreover, the A-ADORA2A complex inhibits the degradation of **ELN**[71] while **MMP9** is involved in upregulating the expression of another collagen, **COL18A1**[76]. **Fibulin 5 (FBLN5)** and **lysyl oxydase oxidase like 1 (LOXL1)** preferentially bind each other[77-79] and the **FBLN5**-**LOXL1** complex facilitates the crosslinking of elastic fibers through binding and interacting with **ELN**[77]. In addition, **lysyl oxidase (LOX)** catalyzes the crosslinking and formation of elastic fibers through **ELN** binding[80-82].

Apart from elastic fiber crosslinking, **LOX** is essential for binding and hence crosslinking collagens such as **COL1A1** and **COL3A1**[83]. **FBLN5** inhibits EMT through significantly enhancing the ability of **TGFB1** to downregulate the expression of **CDH1**[84](not shown). **FBLN5** also increases the expression of **MMP2**[84] and regulates the activity of **MMP9**[84,85]. **MMP2** is a key MMP in the landscape as it degrades **COL1A1**[74] and **ELN**[86] and is downregulated through estradiol-bound **ESR2**[87] and upregulated by NFKB[88]. Further, **MMP2** is involved in upregulating the other MMPs **MMP1**, **MMP3** - which promotes EMT through degrading **CDH1**[89,90] - and **MMP9**[91].

The extracellular protein **TIMP2** binds and forms a complex with **MMP1**[92] and **MMP2**[93] while it inhibits **MMP1**[94], **MMP2**[95] and **MMP9**[96]. **TIMP1** - a protein that is functionally related to **TIMP2** - inhibits **MMP1**[92], **MMP2**[97], **MMP3**[27,98] and **MMP9**[97], while its expression is upregulated by **TGFB1**[99]. Moreover, **MMP3** activates both **MMP9**[100] and **MMP1**[101], with the latter being upregulated by **ELN**[102]. Both **MMP1** and **MMP3** also upregulate the expression of **MMP9**[103]. Additionally, **COL18A1** downregulates the expression of **MMP1**[104] and inhibits

MMP2[105], which itself is involved in upregulating **COL18A1** expression[106]. Further, **BMP1** is an extracellular matrix metalloproteinase that cleaves and regulates the activity of **LOX**[107]. Likewise, **BMP1** directly binds, interacts with and regulates the activity of **COL1A1** through cleavage[107-109]. Also, **FBLN3** downregulates the expression of **FBLN5**[110] while it negatively regulates **MMP2** and **MMP9**[110]. Lastly, **LAIR2** is another ECM protein that binds and functionally interacts with **COL1A1**, **COL3A1** and **COL18A1**[111].

TGFB1 is involved in ECM remodeling through regulating the expression of the majority of the above described ECM proteins. **TGFB1** mediates collagen and elastin fiber deposition in the ECM through (up)regulating the expression of **BMP1**[112,113], **COL1A1**[114-117], **COL3A1**[116], **ECM1**[15], **ELN**[117], **FBLN5**[84,118], **LOX** and **LOXL1**[117]. Moreover, **TGFB1** upregulates the expression of **MMP2**[119], **MMP3**[119,120] and **MMP9**[119,121] while it is involved in decreasing the expression of **MMP1**[122,123]. In addition, cell surface-localized **MMP2** and **MMP9** can cleave and hence activate latent **TGFB1** (not shown)[124].

The extracellular proenzyme **plasminogen** (**PLG**) is cleaved by the PLAU and PLAT enzymes (not shown) to plasmin (PL)[27], an extracellular enzyme that dissolves the fibrin of blood clots and acts as a proteolytic factor in a variety of other biological processes, including ECM remodeling[27]. PLAU binding to its receptor (not shown), **PLAUR**, which is anchored in the fibroblast cell membrane and has a role in regulating fibroblast growth[125], promotes the cleavage of **PLG** to PL[27], a process that is also stimulated by **TGFB1**[126]. PL upregulates the expression of **COL1A1**[127] and it activates **MMP1**, **MMP2**, **MMP3** and **MMP9**[128-130]. Further, PL is inhibited by the extracellular proteinase inhibitor **SERPINA1**, which itself is degraded by **MMP1** (not shown), **MMP2**, **MMP3**, **MMP8** and **MMP9**[131]. The extracellular form of **YWHAB** (see below) is also involved in upregulating the expression of **MMP1**[132].

Since fibroblasts are responsible for the synthesis and secretion of the main ECM components through (e.g. **AHNAK**- or **TGFB1**-activated) **SMAD3** - which also plays a major role in EMT (see above) - fibroblast survival and apoptosis (and hence proper functioning) is another important landscape process. In this respect, **SMAD3** upregulates the expression of **COL1A1**, **COL3A1**[116] and **MMP9**[133], whereas it downregulates **ELN**[134] and **MMP1**[123]. Further, nuclear SPOP belongs to an E3 ubiquitin-protein ligase complex that is involved in upregulating **MMP1** expression[135].

This SPOP complex also represses the transcriptional activity of **ESR1**[136] and its activity is regulated through binding and interacting with **SPOPL**[137].

TRAIL (other name: **TNFSF10**) is a member of the tumor necrosis factor (TNF) family of extracellular cytokines that binds to its receptors, the transmembrane proteins TRAIL-R1 (other name: TNFRSF10A), TRAIL-R2 (other name: TNFRSF10B) and **TRAIL-R3** (other name: **TNFRSF10C**), which is anchored in the fibroblast cell membrane[27]. Upon binding TRAIL-R1[138] and TRAIL-R2[139], **TRAIL** triggers fibroblast apoptosis through activating **CASP3** or NFKB[140,141]. In contrast, **TRAIL-R3** competes with TRAIL-R1 and TRAIL-R2 for **TRAIL** binding and, as **TRAIL-R3** does not have a cytoplasmic 'death domain', it inhibits the TRAIL-R1/2-induced apoptotic cascades and hence protects fibroblasts against apoptosis[142]. **TGFB1** (up)regulates the expression of TRAIL-R1 and **CASP3**[143] and, as already mentioned above, NFKB is activated downstream of **TGFB1** signalling[19]. In turn, NFKB regulates the activity of **CASP3**[144,145], providing additional regulation of the TRAIL-R1/R2-apoptotic signaling cascade. In addition, **TGFB1** promotes EMT through activating miR-155[146], a microRNA that regulates inflammation and fibrosis and is involved in inhibiting **CASP3**[147] and upregulating the expression of **SMAD3**[148]. **CASP3** also cleaves (and hence regulates the activity of) **AHNAK**[149], which, as already indicated above, shuttles between the cytoplasm and nucleus[9] and stimulates the **TGFB1**-induced activation and translocation of **SMAD3** to the nucleus[10]. Further, **CASP3** (negatively) regulates the activity of **PARP1**[150,151], a nuclear protein that modulates oxidative stress-induced apoptosis in fibroblasts[152]. Moreover, **PARP1** is activated by **TRAIL**[153]. **RAD52**, a nuclear DNA repair protein that promotes the survival of fibroblasts[154], also hyperactivates **PARP1**[155]. Further, both **SMAD3**[156] and **YWHAB**[157] bind and interact with **WNK1**, a kinase that is also upregulated by **TGFB1**[158].

In addition, **COL18A1** decreases the expression of **HIF1A**[159], a transcription factor that counteracts apoptosis of fibroblasts[159]. Further, **HIF1A** binds and interacts with **ESR1**[24], **SMAD3**[160] and SPOP[161], while **TGFB1** and **HIF1A** upregulate each other's expression[162,163]. **HIF1A** is also bound and activated by **PARP1**[164], and it is involved in increasing the expression of **MMP1**[165], **MMP2**[166], **MMP9**[166], **LOX** (not shown)[167] and **PLAUR**[167]. Moreover, **NACA2** is a nuclear protein that forms a functional complex with NACA[168], a positive regulator of **COL1A1** expression[169].

Another important protein in the landscape is **vimentin (VIM)**, a cytoskeletal protein of which the expression is increased in mesenchymal cells such as fibroblasts during EMT and that regulates fibroblast function, in particular the response to mechanical stress[170]. **VIM** expression is upregulated by **ECM1**[47] and downregulated by **FBLN3**[51]. while the protein is also cleaved by **CASP3** (not shown)[171] and it is involved in increasing the production of **TGFB1** by fibroblasts[172]. **VIM** expression is also downregulated by **TBX5**[173], a nuclear transcription factor that is found in fibroblasts[174] and also downregulates the expression of both **COL1A1**[175] and **COL1A3**[175]. Moreover, **VIM** upregulates the expression of **ITGB1**[176], a membrane protein of the integrin family that downregulates **COL1A1** expression[177], is involved in regulating the activity of **COL18A1**[178] and is upregulated downstream of both **TGFB1**[158] and **PLAUR**[179]. **ITGB1** upregulates the expression of **MMP9** - which is inhibited by **FBLN5**[180] - and of **LAMC1**[181], an ECM protein that binds and is upregulated by **TGFB1**[182,183]. **ITGB1** is also bound and regulated by **EMILIN1**[184,185], an ECM proteins that is secreted by fibroblasts[186] and binds/interacts with both **ELN**[187] and **LAIR2** [188]. Further, **EMILIN1** inhibits **TGFB1** signaling[189].

As already indicated above, the **AGE**-**RAGE** complex regulates a number of downstream signaling cascades in both epithelial cells and fibroblasts. In this respect, the **AGE**-**RAGE** complex stimulates the apoptosis of fibroblasts[190] in which it regulates the activity of NFKB[21,191] and inhibits **CASP3**[192]. Further, **AGE**-**RAGE** is involved in upregulating the expression of **MMP2**[193], **MMP9**[194] - which is itself inhibited by **ECM1**[195] - and **TGFB1**[196], while it also promotes the translocation of **SMAD3** from the cytoplasm to the nucleus of fibroblasts[21].

Lastly, in fibroblasts, **MRPL35** is located in and affects the function of mitochondria through regulating the synthesis of the cytochrome C oxidase (COX) enzyme (not shown), which is part of the IVth respiratory chain complex[197] that, together with other respiratory chain complexes, may be expressed at lower levels in women with POP[198].

References

1. Lamouille S, Xu J, Derynck R. Molecular mechanisms of epithelial-mesenchymal transition. *Nat Rev Mol Cell Biol.* 2014;15(3):178-196.
2. Davis TA, Loos B, Engelbrecht AM. AHNAK: the giant jack of all trades. *Cell Signal.* 2014;26(12):2683-2693.
3. Benaud C, Gentil BJ, Assard N, et al. AHNAK interaction with the annexin 2/S100A10 complex regulates cell membrane cytoarchitecture. *J Cell Biol.* 2004;164(1):133-144.
4. McElhinny AS, Kazmierski ST, Labeit S, Gregorio CC. Nebulin: the nebulous, multifunctional giant of striated muscle. *Trends Cardiovasc Med.* 2003;13(5):195-201.
5. Caddy J, Wilanowski T, Darido C, et al. Epidermal wound repair is regulated by the planar cell polarity signaling pathway. *Dev Cell.* 2010;19(1):138-147.
6. Ishikawa F, Nose K, Shibanuma M. Downregulation of hepatocyte nuclear factor-4alpha and its role in regulation of gene expression by TGF-beta in mammary epithelial cells. *Exp Cell Res.* 2008;314(10):2131-2140.
7. Shan B, Yao TP, Nguyen HT, et al. Requirement of HDAC6 for transforming growth factor-beta1-induced epithelial-mesenchymal transition. *J Biol Chem.* 2008;283(30):21065-21073.
8. Lin YC, Lee YC, Li LH, Cheng CJ, Yang RB. Tumor suppressor SCUBE2 inhibits breast-cancer cell migration and invasion through the reversal of epithelial-mesenchymal transition. *J Cell Sci.* 2014;127(Pt 1):85-100.
9. Sussman J, Stokoe D, Ossina N, Shtivelman E. Protein kinase B phosphorylates AHNAK and regulates its subcellular localization. *J Cell Biol.* 2001;154(5):1019-1030.
10. Lee IH, Sohn M, Lim HJ, et al. Ahnak functions as a tumor suppressor via modulation of TGFbeta/Smad signaling pathway. *Oncogene.* 2014;33(38):4675-4684.
11. Tsukazaki T, Chiang TA, Davison AF, Attisano L, Wrana JL. SARA, a FYVE domain protein that recruits Smad2 to the TGFbeta receptor. *Cell.* 1998;95(6):779-791.
12. Qin BY, Lam SS, Correia JJ, Lin K. Smad3 allostery links TGF-beta receptor kinase activation to transcriptional control. *Genes Dev.* 2002;16(15):1950-1963.
13. Colland F, Jacq X, Trouplin V, et al. Functional proteomics mapping of a human signaling pathway. *Genome Res.* 2004;14(7):1324-1332.
14. Runyan CE, Hayashida T, Hubchak S, Curley JF, Schnaper HW. Role of SARA (SMAD anchor for receptor activation) in maintenance of epithelial cell phenotype. *J Biol Chem.* 2009;284(37):25181-25189.
15. Luo X, Ding L, Xu J, Chegini N. Gene expression profiling of leiomyoma and myometrial smooth muscle cells in response to transforming growth factor-beta. *Endocrinology.* 2005;146(3):1097-1118.
16. Xie W, Huang Y, Xie W, Guo A, Wu W. Bacteria peptidoglycan promoted breast cancer cell invasiveness and adhesiveness by targeting toll-like receptor 2 in the cancer cells. *PLoS One.* 2010;5(5):e10850.
17. Gewurz BE, Towfic F, Mar JC, et al. Genome-wide siRNA screen for mediators of NF-kappaB activation. *Proc Natl Acad Sci U S A.* 2012;109(7):2467-2472.
18. Duxbury MS, Whang EE. RRM2 induces NF-kappaB-dependent MMP-9 activation and enhances cellular invasiveness. *Biochem Biophys Res Commun.* 2007;354(1):190-196.
19. Sakuma M, Hatsushika K, Koyama K, et al. TGF-beta type I receptor kinase inhibitor down-regulates rheumatoid synoviocytes and prevents the arthritis induced by type II collagen antibody. *Int Immunol.* 2007;19(2):117-126.
20. Sato M, Muragaki Y, Saika S, Roberts AB, Ooshima A. Targeted disruption of TGF-beta1/Smad3 signaling protects against renal tubulointerstitial fibrosis induced by unilateral ureteral obstruction. *J Clin Invest.* 2003;112(10):1486-1494.
21. Lan HY. Diverse roles of TGF-beta/Smads in renal fibrosis and inflammation. *Int J Biol Sci.* 2011;7(7):1056-1067.
22. Tomic D, Miller KP, Kenny HA, Woodruff TK, Hoyer P, Flaws JA. Ovarian follicle development requires Smad3. *Mol Endocrinol.* 2004;18(9):2224-2240.
23. de Leeuw R, Flach K, Bentin Toaldo C, et al. PKA phosphorylation redirects ERalpha to promoters of a unique gene set to induce tamoxifen resistance. *Oncogene.* 2013;32(30):3543-3551.
24. Tarallo R, Bamundo A, Nassa G, et al. Identification of proteins associated with ligand-activated estrogen receptor alpha in human breast cancer cell nuclei by tandem affinity purification and nano LC-MS/MS. *Proteomics.* 2011;11(1):172-179.
25. Zhao JJ, Lin J, Yang H, et al. MicroRNA-221/222 negatively regulates estrogen receptor alpha and is associated with tamoxifen resistance in breast cancer. *J Biol Chem.* 2008;283(45):31079-31086.

26. Hayano T, Yanagida M, Yamauchi Y, Shinkawa T, Isobe T, Takahashi N. Proteomic analysis of human Nop56p-associated pre-ribosomal ribonucleoprotein complexes. Possible link between Nop56p and the nucleolar protein treacle responsible for Treacher Collins syndrome. *J Biol Chem.* 2003;278(36):34309-34319.
27. UniProt C. UniProt: a worldwide hub of protein knowledge. *Nucleic Acids Res.* 2019;47(D1):D506-D515.
28. Milojevic T, Reiterer V, Stefan E, et al. The ubiquitin-specific protease Usp4 regulates the cell surface level of the A2A receptor. *Mol Pharmacol.* 2006;69(4):1083-1094.
29. Garcia GE, Truong LD, Chen JF, Johnson RJ, Feng L. Adenosine A(2A) receptor activation prevents progressive kidney fibrosis in a model of immune-associated chronic inflammation. *Kidney Int.* 2011;80(4):378-388.
30. Xiao H, Shen HY, Liu W, et al. Adenosine A2A receptor: a target for regulating renal interstitial fibrosis in obstructive nephropathy. *PLoS One.* 2013;8(4):e60173.
31. Shi Y, Zhang J, Liu M, Huang Y, Yin L. SMAD3 inducing the transcription of STYK1 to promote the EMT process and improve the tolerance of ovarian carcinoma cells to paclitaxel. *J Cell Biochem.* 2019;120(6):10796-10811.
32. Wang Z, Qu L, Deng B, et al. STYK1 promotes epithelial-mesenchymal transition and tumor metastasis in human hepatocellular carcinoma through MEK/ERK and PI3K/AKT signaling. *Sci Rep.* 2016;6:33205.
33. Weihua Z, Saji S, Makinen S, et al. Estrogen receptor (ER) beta, a modulator of ERalpha in the uterus. *Proc Natl Acad Sci U S A.* 2000;97(11):5936-5941.
34. Jeong JW, Lee KY, Kwak I, et al. Identification of murine uterine genes regulated in a ligand-dependent manner by the progesterone receptor. *Endocrinology.* 2005;146(8):3490-3505.
35. Binder AK, Rodriguez KF, Hamilton KJ, Stockton PS, Reed CE, Korach KS. The absence of ER-beta results in altered gene expression in ovarian granulosa cells isolated from in vivo preovulatory follicles. *Endocrinology.* 2013;154(6):2174-2187.
36. Oakley AJ, Yamada T, Liu D, Coggan M, Clark AG, Board PG. The identification and structural characterization of C7orf24 as gamma-glutamyl cyclotransferase. An essential enzyme in the gamma-glutamyl cycle. *J Biol Chem.* 2008;283(32):22031-22042.
37. Amano T, Eishi Y, Yamada T, et al. Widespread expression of gamma-glutamyl cyclotransferase suggests it is not a general tumor marker. *J Histochem Cytochem.* 2012;60(1):76-86.
38. Frottin F, Bienvenut WV, Bignon J, et al. MetAP1 and MetAP2 drive cell selectivity for a potent anti-cancer agent in synergy, by controlling glutathione redox state. *Oncotarget.* 2016;7(39):63306-63323.
39. Gefter JV, Shaufl AL, Fink MP, Delude RL. Comparison of distinct protein isoforms of the receptor for advanced glycation end-products expressed in murine tissues and cell lines. *Cell Tissue Res.* 2009;337(1):79-89.
40. Lewis P, Stefanovic N, Pete J, et al. Lack of the antioxidant enzyme glutathione peroxidase-1 accelerates atherosclerosis in diabetic apolipoprotein E-deficient mice. *Circulation.* 2007;115(16):2178-2187.
41. Henderson CJ, Ritchie KJ, McLaren A, Chakravarty P, Wolf CR. Increased skin papilloma formation in mice lacking glutathione transferase GSTP. *Cancer Res.* 2011;71(22):7048-7060.
42. Lerner M, Lemke D, Bertram H, et al. An extracellular loop of the human non-gastric H,K-ATPase alpha-subunit is involved in apical plasma membrane polarization. *Cell Physiol Biochem.* 2006;18(1-3):75-84.
43. Ye Y, Xiao Y, Wang W, et al. ERalpha signaling through slug regulates E-cadherin and EMT. *Oncogene.* 2010;29(10):1451-1462.
44. Forster C, Makela S, Warri A, et al. Involvement of estrogen receptor beta in terminal differentiation of mammary gland epithelium. *Proc Natl Acad Sci U S A.* 2002;99(24):15578-15583.
45. Guo B, Han BC, Tian Z, et al. Expression and hormonal regulation of E-cadherin in canine uterus during early pregnancy. *Reprod Domest Anim.* 2010;45(6):e255-259.
46. Lin VC, Jin R, Tan PH, Aw SE, Woon CT, Bay BH. Progesterone induces cellular differentiation in MDA-MB-231 breast cancer cells transfected with progesterone receptor complementary DNA. *Am J Pathol.* 2003;162(6):1781-1787.
47. Lee KM, Nam K, Oh S, et al. ECM1 regulates tumor metastasis and CSC-like property through stabilization of beta-catenin. *Oncogene.* 2015;34(50):6055-6065.
48. Li Y, Hamilton KJ, Wang T, et al. DNA methylation and transcriptome aberrations mediated by ERalpha in mouse seminal vesicles following developmental DES exposure. *Proc Natl Acad Sci U S A.* 2018;115(18):E4189-E4198.
49. Guo Y, Li Z, Ding R, et al. Parathyroid hormone induces epithelial-to-mesenchymal transition via the Wnt/beta-catenin signaling pathway in human renal proximal tubular cells. *Int J Clin Exp Pathol.* 2014;7(9):5978-5987.
50. Paul SM, Halaris AE. Rat brain de-acetylating activity: stereospecific inhibition by LSD and serotonin-related compounds. *Biochem Biophys Res Commun.* 1976;70(1):207-211.
51. Yang T, Qiu H, Bao W, et al. Epigenetic inactivation of EFEMP1 is associated with tumor suppressive function in endometrial carcinoma. *PLoS One.* 2013;8(6):e67458.

52. Tian H, Liu J, Chen J, Gatza ML, Blobe GC. Fibulin-3 is a novel TGF-beta pathway inhibitor in the breast cancer microenvironment. *Oncogene.* 2015;34(45):5635-5647.
53. Aspuria PP, Lunt SY, Varemo L, et al. Succinate dehydrogenase inhibition leads to epithelial-mesenchymal transition and reprogrammed carbon metabolism. *Cancer Metab.* 2014;2:21.
54. Garrido N, Griparic L, Jokitalo E, Wartiovaara J, van der Bliek AM, Spelbrink JN. Composition and dynamics of human mitochondrial nucleoids. *Mol Biol Cell.* 2003;14(4):1583-1596.
55. Sun MJ, Cheng WL, Wei YH, et al. Low copy number and high 4977 deletion of mitochondrial DNA in uterosacral ligaments are associated with pelvic organ prolapse progression. *Int Urogynecol J Pelvic Floor Dysfunct.* 2009;20(7):867-872.
56. Sun MJ, Cheng YS, Sun R, Cheng WL, Liu CS. Changes in mitochondrial DNA copy number and extracellular matrix (ECM) proteins in the uterosacral ligaments of premenopausal women with pelvic organ prolapse. *Taiwan J Obstet Gynecol.* 2016;55(1):9-15.
57. Sun MJ, Cheng YS, Liu CS, Sun R. Changes in the PGC-1alpha and mtDNA copy number may play a role in the development of pelvic organ prolapse in pre-menopausal patients. *Taiwan J Obstet Gynecol.* 2019;58(4):526-530.
58. Josson S, Nomura T, Lin JT, et al. beta2-microglobulin induces epithelial to mesenchymal transition and confers cancer lethality and bone metastasis in human cancer cells. *Cancer Res.* 2011;71(7):2600-2610.
59. Nomura T, Huang WC, Zhau HE, Josson S, Mimata H, Chung LW. beta2-Microglobulin-mediated signaling as a target for cancer therapy. *Anticancer Agents Med Chem.* 2014;14(3):343-352.
60. Ju W, Eichinger F, Bitzer M, et al. Renal gene and protein expression signatures for prediction of kidney disease progression. *Am J Pathol.* 2009;174(6):2073-2085.
61. Allen RL, Raine T, Haude A, Trowsdale J, Wilson MJ. Leukocyte receptor complex-encoded immunomodulatory receptors show differing specificity for alternative HLA-B27 structures. *J Immunol.* 2001;167(10):5543-5547.
62. Kobayashi S, Yoshida K, Ward JM, et al. Beta 2-microglobulin-deficient background ameliorates lethal phenotype of the TGF-beta 1 null mouse. *J Immunol.* 1999;163(7):4013-4019.
63. Reinherz EL, Tan K, Tang L, et al. The crystal structure of a T cell receptor in complex with peptide and MHC class II. *Science.* 1999;286(5446):1913-1921.
64. Wallace PK, Yeaman GR, Johnson K, Collins JE, Guyre PM, Wira CR. MHC class II expression and antigen presentation by human endometrial cells. *J Steroid Biochem Mol Biol.* 2001;76(1-5):203-211.
65. Ljunggren G, Anderson DJ. Cytokine induced modulation of MHC class I and class II molecules on human cervical epithelial cells. *J Reprod Immunol.* 1998;38(2):123-138.
66. Ruiz-Zapata AM, Kerkhof MH, Zandieh-Doulabi B, Brolmann HA, Smit TH, Helder MN. Fibroblasts from women with pelvic organ prolapse show differential mechanoresponses depending on surface substrates. *Int Urogynecol J.* 2013;24(9):1567-1575.
67. Ruiz-Zapata AM, Kerkhof MH, Zandieh-Doulabi B, Brolmann HA, Smit TH, Helder MN. Functional characteristics of vaginal fibroblastic cells from premenopausal women with pelvic organ prolapse. *Mol Hum Reprod.* 2014;20(11):1135-1143.
68. Ewies AA, Elshafie M, Li J, et al. Changes in transcription profile and cytoskeleton morphology in pelvic ligament fibroblasts in response to stretch: the effects of estradiol and levormeloxifene. *Mol Hum Reprod.* 2008;14(2):127-135.
69. Che J, Chan ES, Cronstein BN. Adenosine A2A receptor occupancy stimulates collagen expression by hepatic stellate cells via pathways involving protein kinase A, Src, and extracellular signal-regulated kinases 1/2 signaling cascade or p38 mitogen-activated protein kinase signaling pathway. *Mol Pharmacol.* 2007;72(6):1626-1636.
70. Perez-Aso M, Fernandez P, Mediero A, Chan ES, Cronstein BN. Adenosine 2A receptor promotes collagen production by human fibroblasts via pathways involving cyclic AMP and AKT but independent of Smad2/3. *FASEB J.* 2014;28(2):802-812.
71. Bhamidipati CM, Mehta GS, Moehle CW, et al. Adenosine 2A receptor modulates inflammation and phenotype in experimental abdominal aortic aneurysms. *FASEB J.* 2013;27(6):2122-2131.
72. Lee Y, Kim H, Kim S, Kim KH, Chung JH. Activation of toll-like receptors 2, 3 or 5 induces matrix metalloproteinase-1 and -9 expression with the involvement of MAPKs and NF-kappaB in human epidermal keratinocytes. *Exp Dermatol.* 2010;19(8):e44-49.
73. Tobar N, Villar V, Santibanez JF. ROS-NFkappaB mediates TGF-beta1-induced expression of urokinase-type plasminogen activator, matrix metalloproteinase-9 and cell invasion. *Mol Cell Biochem.* 2010;340(1-2):195-202.
74. LeBleu VS, Teng Y, O'Connell JT, et al. Identification of human epididymis protein-4 as a fibroblast-derived mediator of fibrosis. *Nat Med.* 2013;19(2):227-231.

75. Murphy G, Cockett MI, Ward RV, Docherty AJ. Matrix metalloproteinase degradation of elastin, type IV collagen and proteoglycan. A quantitative comparison of the activities of 95 kDa and 72 kDa gelatinases, stromelysins-1 and -2 and punctuated metalloproteinase (PUMP). *Biochem J.* 1991;277 (Pt 1):277-279.
76. Tolstanova G, Deng X, Khomenko T, et al. Role of anti-angiogenic factor endostatin in the pathogenesis of experimental ulcerative colitis. *Life Sci.* 2011;88(1-2):74-81.
77. Papke CL, Yanagisawa H. Fibulin-4 and fibulin-5 in elastogenesis and beyond: Insights from mouse and human studies. *Matrix Biol.* 2014;37:142-149.
78. Northington GM. Fibulin-5: two for the price of one maintaining pelvic support. *J Clin Invest.* 2011;121(5):1688-1691.
79. Liu X, Zhao Y, Gao J, et al. Elastic fiber homeostasis requires lysyl oxidase-like 1 protein. *Nat Genet.* 2004;36(2):178-182.
80. Kerkhof MH, Hendriks L, Brolmann HA. Changes in connective tissue in patients with pelvic organ prolapse--a review of the current literature. *Int Urogynecol J Pelvic Floor Dysfunct.* 2009;20(4):461-474.
81. Papachroni KK, Piperi C, Levidou G, et al. Lysyl oxidase interacts with AGE signalling to modulate collagen synthesis in polycystic ovarian tissue. *J Cell Mol Med.* 2010;14(10):2460-2469.
82. Hornstra IK, Birge S, Starcher B, Bailey AJ, Mecham RP, Shapiro SD. Lysyl oxidase is required for vascular and diaphragmatic development in mice. *J Biol Chem.* 2003;278(16):14387-14393.
83. Sanchez-Morgan N, Kirsch KH, Trackman PC, Sonenshein GE. The lysyl oxidase propeptide interacts with the receptor-type protein tyrosine phosphatase kappa and inhibits beta-catenin transcriptional activity in lung cancer cells. *Mol Cell Biol.* 2011;31(16):3286-3297.
84. Lee YH, Albig AR, Regner M, Schiemann BJ, Schiemann WP. Fibulin-5 initiates epithelial-mesenchymal transition (EMT) and enhances EMT induced by TGF-beta in mammary epithelial cells via a MMP-dependent mechanism. *Carcinogenesis.* 2008;29(12):2243-2251.
85. Khadzhieva MB, Kolobkov DS, Kamoeva SV, Salnikova LE. Expression changes in pelvic organ prolapse: a systematic review and in silico study. *Sci Rep.* 2017;7(1):7668.
86. Senior RM, Griffin GL, Fliszar CJ, Shapiro SD, Goldberg GI, Welgus HG. Human 92- and 72-kilodalton type IV collagenases are elastases. *J Biol Chem.* 1991;266(12):7870-7875.
87. Morani A, Barros RP, Imamov O, et al. Lung dysfunction causes systemic hypoxia in estrogen receptor beta knockout (ERbeta-/-) mice. *Proc Natl Acad Sci U S A.* 2006;103(18):7165-7169.
88. Choi JY, Piao MS, Lee JB, Oh JS, Kim IG, Lee SC. Propionibacterium acnes stimulates pro-matrix metalloproteinase-2 expression through tumor necrosis factor-alpha in human dermal fibroblasts. *J Invest Dermatol.* 2008;128(4):846-854.
89. Lochter A, Galosy S, Muschler J, Freedman N, Werb Z, Bissell MJ. Matrix metalloproteinase stromelysin-1 triggers a cascade of molecular alterations that leads to stable epithelial-to-mesenchymal conversion and a premalignant phenotype in mammary epithelial cells. *J Cell Biol.* 1997;139(7):1861-1872.
90. Noe V, Fingleton B, Jacobs K, et al. Release of an invasion promoter E-cadherin fragment by matrilysin and stromelysin-1. *J Cell Sci.* 2001;114(Pt 1):111-118.
91. Ehrentraut H, Meyer R, Schwederski M, et al. Systemically administered ligands of Toll-like receptor 2, -4, and -9 induce distinct inflammatory responses in the murine lung. *Mediators Inflamm.* 2011;2011:746532.
92. Bahudhanapati H, Zhang Y, Sidhu SS, Brew K. Phage display of tissue inhibitor of metalloproteinases-2 (TIMP-2): identification of selective inhibitors of collagenase-1 (metalloproteinase 1 (MMP-1)). *J Biol Chem.* 2011;286(36):31761-31770.
93. Nissi R, Talvensaari-Mattila A, Kotila V, Niinimaki M, Jarvela I, Turpeenniemi-Hujanen T. Circulating matrix metalloproteinase MMP-9 and MMP-2/TIMP-2 complex are associated with spontaneous early pregnancy failure. *Reprod Biol Endocrinol.* 2013;11:2.
94. Bataller R, Brenner DA. Hepatic stellate cells as a target for the treatment of liver fibrosis. *Semin Liver Dis.* 2001;21(3):437-451.
95. Mroczko B, Lukaszewicz-Zajac M, Gryko M, Kedra B, Szmitkowski M. Clinical significance of serum levels of matrix metalloproteinase 2 (MMP-2) and its tissue inhibitor (TIMP-2) in gastric cancer. *Folia Histochem Cytobiol.* 2011;49(1):125-131.
96. Zhao YG, Xiao AZ, Park HI, et al. Endometase/matrilysin-2 in human breast ductal carcinoma in situ and its inhibition by tissue inhibitors of metalloproteinases-2 and -4: a putative role in the initiation of breast cancer invasion. *Cancer Res.* 2004;64(2):590-598.
97. Nadeem L, Munir S, Fu G, et al. Nodal signals through activin receptor-like kinase 7 to inhibit trophoblast migration and invasion: implication in the pathogenesis of preeclampsia. *Am J Pathol.* 2011;178(3):1177-1189.

98. Kim EM, Shin EJ, Choi JH, et al. Matrix metalloproteinase-3 is increased and participates in neuronal apoptotic signaling downstream of caspase-12 during endoplasmic reticulum stress. *J Biol Chem.* 2010;285(22):16444-16452.
99. Overall CM. Repression of tissue inhibitor of matrix metalloproteinase expression by all-trans-retinoic acid in rat bone cell populations: comparison with transforming growth factor-beta 1. *J Cell Physiol.* 1995;164(1):17-25.
100. Rigot V, Marbaix E, Lemoine P, Courtoy PJ, Eeckhout Y. In vivo perimenstrual activation of progelatinase B (proMMP-9) in the human endometrium and its dependence on stromelysin 1 (MMP-3) ex vivo. *Biochem J.* 2001;358(Pt 1):275-280.
101. Visse R, Nagase H. Matrix metalloproteinases and tissue inhibitors of metalloproteinases: structure, function, and biochemistry. *Circ Res.* 2003;92(8):827-839.
102. Brassart B, Fuchs P, Huet E, et al. Conformational dependence of collagenase (matrix metalloproteinase-1) up-regulation by elastin peptides in cultured fibroblasts. *J Biol Chem.* 2001;276(7):5222-5227.
103. Steenport M, Khan KM, Du B, Barnhard SE, Dannenberg AJ, Falcone DJ. Matrix metalloproteinase (MMP)-1 and MMP-3 induce macrophage MMP-9: evidence for the role of TNF-alpha and cyclooxygenase-2. *J Immunol.* 2009;183(12):8119-8127.
104. Abdollahi A, Hahnfeldt P, Maercker C, et al. Endostatin's antiangiogenic signaling network. *Mol Cell.* 2004;13(5):649-663.
105. Egeblad M, Werb Z. New functions for the matrix metalloproteinases in cancer progression. *Nat Rev Cancer.* 2002;2(3):161-174.
106. Deng X, Xiong X, Khomenko T, et al. Inappropriate angiogenic response as a novel mechanism of duodenal ulceration and impaired healing. *Dig Dis Sci.* 2011;56(10):2792-2801.
107. Uzel MI, Scott IC, Babakhanlou-Chase H, et al. Multiple bone morphogenetic protein 1-related mammalian metalloproteinases process pro-lysyl oxidase at the correct physiological site and control lysyl oxidase activation in mouse embryo fibroblast cultures. *J Biol Chem.* 2001;276(25):22537-22543.
108. von Marschall Z, Fisher LW. Dentin sialophosphoprotein (DSPP) is cleaved into its two natural dentin matrix products by three isoforms of bone morphogenetic protein-1 (BMP1). *Matrix Biol.* 2010;29(4):295-303.
109. Oganesian A, Au S, Horst JA, et al. The NH2-terminal propeptide of type I procollagen acts intracellularly to modulate cell function. *J Biol Chem.* 2006;281(50):38507-38518.
110. Rahn DD, Acevedo JF, Roshanravan S, et al. Failure of pelvic organ support in mice deficient in fibulin-3. *Am J Pathol.* 2009;174(1):206-215.
111. Lebbink RJ, van den Berg MC, de Ruiter T, et al. The soluble leukocyte-associated Ig-like receptor (LAIR)-2 antagonizes the collagen/LAIR-1 inhibitory immune interaction. *J Immunol.* 2008;180(3):1662-1669.
112. Lee S, Solow-Cordero DE, Kessler E, Takahara K, Greenspan DS. Transforming growth factor-beta regulation of bone morphogenetic protein-1/procollagen C-proteinase and related proteins in fibrogenic cells and keratinocytes. *J Biol Chem.* 1997;272(30):19059-19066.
113. Verga-Gerard A, Porcherot M, Meyniel-Schicklin L, Andre P, Lotteau V, Perrin-Cocon L. Hepatitis C virus/human interactome identifies SMURF2 and the viral protease as critical elements for the control of TGF-beta signaling. *FASEB J.* 2013;27(10):4027-4040.
114. Wilson CA, Cajulis EE, Green JL, et al. HER-2 overexpression differentially alters transforming growth factor-beta responses in luminal versus mesenchymal human breast cancer cells. *Breast Cancer Res.* 2005;7(6):R1058-1079.
115. Chen SJ, Ning H, Ishida W, et al. The early-immediate gene EGR-1 is induced by transforming growth factor-beta and mediates stimulation of collagen gene expression. *J Biol Chem.* 2006;281(30):21183-21197.
116. Verrecchia F, Chu ML, Mauviel A. Identification of novel TGF-beta /Smad gene targets in dermal fibroblasts using a combined cDNA microarray/promoter transactivation approach. *J Biol Chem.* 2001;276(20):17058-17062.
117. Chambers RC, Leoni P, Kaminski N, Laurent GJ, Heller RA. Global expression profiling of fibroblast responses to transforming growth factor-beta1 reveals the induction of inhibitor of differentiation-1 and provides evidence of smooth muscle cell phenotypic switching. *Am J Pathol.* 2003;162(2):533-546.
118. Schiemann WP, Blobe GC, Kalume DE, Pandey A, Lodish HF. Context-specific effects of fibulin-5 (DANCE/EVEC) on cell proliferation, motility, and invasion. Fibulin-5 is induced by transforming growth factor-beta and affects protein kinase cascades. *J Biol Chem.* 2002;277(30):27367-27377.
119. Hawinkels LJ, Paauwe M, Verspaget HW, et al. Interaction with colon cancer cells hyperactivates TGF-beta signaling in cancer-associated fibroblasts. *Oncogene.* 2014;33(1):97-107.
120. Oriente A, Fedarko NS, Pacocha SE, Huang SK, Lichtenstein LM, Essayan DM. Interleukin-13 modulates collagen homeostasis in human skin and keloid fibroblasts. *J Pharmacol Exp Ther.* 2000;292(3):988-994.

121. Saad S, Stanners SR, Yong R, Tang O, Pollock CA. Notch mediated epithelial to mesenchymal transformation is associated with increased expression of the Snail transcription factor. *Int J Biochem Cell Biol.* 2010;42(7):1115-1122.
122. Asano Y, Ihn H, Yamane K, Jinnin M, Mimura Y, Tamaki K. Increased expression of integrin alpha(v)beta3 contributes to the establishment of autocrine TGF-beta signaling in scleroderma fibroblasts. *J Immunol.* 2005;175(11):7708-7718.
123. Yuan W, Varga J. Transforming growth factor-beta repression of matrix metalloproteinase-1 in dermal fibroblasts involves Smad3. *J Biol Chem.* 2001;276(42):38502-38510.
124. Yu Q, Stamenkovic I. Cell surface-localized matrix metalloproteinase-9 proteolytically activates TGF-beta and promotes tumor invasion and angiogenesis. *Genes Dev.* 2000;14(2):163-176.
125. Mazzieri R, Furlan F, D'Alessio S, et al. A direct link between expression of urokinase plasminogen activator receptor, growth rate and oncogenic transformation in mouse embryonic fibroblasts. *Oncogene.* 2007;26(5):725-732.
126. Saksela O, Rifkin DB. Cell-associated plasminogen activation: regulation and physiological functions. *Annu Rev Cell Biol.* 1988;4:93-126.
127. Okunishi K, Sisson TH, Huang SK, Hogaboam CM, Simon RH, Peters-Golden M. Plasmin overcomes resistance to prostaglandin E2 in fibrotic lung fibroblasts by reorganizing protein kinase A signaling. *J Biol Chem.* 2011;286(37):32231-32243.
128. Lijnen HR. Plasmin and matrix metalloproteinases in vascular remodeling. *Thromb Haemost.* 2001;86(1):324-333.
129. Nagase H. Activation mechanisms of matrix metalloproteinases. *Biol Chem.* 1997;378(3-4):151-160.
130. Ferrer MJ, Xu W, Shetty J, Herr J, Oko R. Plasminogen Improves Mouse IVF by Interactions with Inner Acrosomal Membrane-Bound MMP2 and SAMP14. *Biol Reprod.* 2016.
131. Sternlicht MD, Werb Z. How matrix metalloproteinases regulate cell behavior. *Annu Rev Cell Dev Biol.* 2001;17:463-516.
132. Asdaghi N, Kilani RT, Hosseini-Tabatabaei A, et al. Extracellular 14-3-3 from human lung epithelial cells enhances MMP-1 expression. *Mol Cell Biochem.* 2012;360(1-2):261-270.
133. Kim S, Han J, Lee SK, et al. Smad7 acts as a negative regulator of the epidermal growth factor (EGF) signaling pathway in breast cancer cells. *Cancer Lett.* 2012;314(2):147-154.
134. Arany PR, Flanders KC, Kobayashi T, et al. Smad3 deficiency alters key structural elements of the extracellular matrix and mechanotransduction of wound closure. *Proc Natl Acad Sci U S A.* 2006;103(24):9250-9255.
135. Kwon JE, La M, Oh KH, et al. BTB domain-containing speckle-type POZ protein (SPOP) serves as an adaptor of Daxx for ubiquitination by Cul3-based ubiquitin ligase. *J Biol Chem.* 2006;281(18):12664-12672.
136. Byun B, Jung Y. Repression of transcriptional activity of estrogen receptor alpha by a Cullin3/SPOP ubiquitin E3 ligase complex. *Mol Cells.* 2008;25(2):289-293.
137. Errington WJ, Khan MQ, Bueler SA, Rubinstein JL, Chakrabartty A, Prive GG. Adaptor protein self-assembly drives the control of a cullin-RING ubiquitin ligase. *Structure.* 2012;20(7):1141-1153.
138. Pan G, O'Rourke K, Chinnaiyan AM, et al. The receptor for the cytotoxic ligand TRAIL. *Science.* 1997;276(5309):111-113.
139. Walczak H, Degli-Esposti MA, Johnson RS, et al. TRAIL-R2: a novel apoptosis-mediating receptor for TRAIL. *EMBO J.* 1997;16(17):5386-5397.
140. MacFarlane M. TRAIL-induced signalling and apoptosis. *Toxicol Lett.* 2003;139(2-3):89-97.
141. Neumann S, Bidon T, Branschadel M, Krippner-Heidenreich A, Scheurich P, Doszczak M. The transmembrane domains of TNF-related apoptosis-inducing ligand (TRAIL) receptors 1 and 2 co-regulate apoptotic signaling capacity. *PLoS One.* 2012;7(8):e42526.
142. Degli-Esposti MA, Smolak PJ, Walczak H, et al. Cloning and characterization of TRAIL-R3, a novel member of the emerging TRAIL receptor family. *J Exp Med.* 1997;186(7):1165-1170.
143. Jazag A, Ijichi H, Kanai F, et al. Smad4 silencing in pancreatic cancer cell lines using stable RNA interference and gene expression profiles induced by transforming growth factor-beta. *Oncogene.* 2005;24(4):662-671.
144. Eo SH, Cho H, Kim SJ. Resveratrol Inhibits Nitric Oxide-Induced Apoptosis via the NF-Kappa B Pathway in Rabbit Articular Chondrocytes. *Biomol Ther (Seoul).* 2013;21(5):364-370.
145. Langone P, Debata PR, Dolai S, et al. Coupling to a cancer cell-specific antibody potentiates tumoricidal properties of curcumin. *Int J Cancer.* 2012;131(4):E569-578.
146. Liu F, Kong X, Lv L, Gao J. TGF-beta1 acts through miR-155 to down-regulate TP53INP1 in promoting epithelial-mesenchymal transition and cancer stem cell phenotypes. *Cancer Lett.* 2015;359(2):288-298.

147. Ovcharenko D, Kelnar K, Johnson C, Leng N, Brown D. Genome-scale microRNA and small interfering RNA screens identify small RNA modulators of TRAIL-induced apoptosis pathway. *Cancer Res.* 2007;67(22):10782-10788.
148. Csak T, Bala S, Lippai D, et al. MicroRNA-155 Deficiency Attenuates Liver Steatosis and Fibrosis without Reducing Inflammation in a Mouse Model of Steatohepatitis. *PLoS One.* 2015;10(6):e0129251.
149. Skoldberg F, Ronnblom L, Thornemo M, et al. Identification of AHNAK as a novel autoantigen in systemic lupus erythematosus. *Biochem Biophys Res Commun.* 2002;291(4):951-958.
150. Tafani M, Karpinich NO, Hurster KA, et al. Cytochrome c release upon Fas receptor activation depends on translocation of full-length bid and the induction of the mitochondrial permeability transition. *J Biol Chem.* 2002;277(12):10073-10082.
151. Boulares AH, Yakovlev AG, Ivanova V, et al. Role of poly(ADP-ribose) polymerase (PARP) cleavage in apoptosis. Caspase 3-resistant PARP mutant increases rates of apoptosis in transfected cells. *J Biol Chem.* 1999;274(33):22932-22940.
152. Kim JY, Kim EJ, Jeon MJ, Kim H, Moon YJ, Bai SW. Association between the poly(ADP-ribose) polymerase-1 gene polymorphism and advanced pelvic organ prolapse. *Menopause.* 2014;21(2):177-181.
153. Xu J, Zhou JY, Wei WZ, Philipsen S, Wu GS. Sp1-mediated TRAIL induction in chemosensitization. *Cancer Res.* 2008;68(16):6718-6726.
154. Feng Z, Scott SP, Bussen W, et al. Rad52 inactivation is synthetically lethal with BRCA2 deficiency. *Proc Natl Acad Sci U S A.* 2011;108(2):686-691.
155. Gottipati P, Vischioni B, Schultz N, et al. Poly(ADP-ribose) polymerase is hyperactivated in homologous recombination-defective cells. *Cancer Res.* 2010;70(13):5389-5398.
156. Lee BH, Chen W, Stippec S, Cobb MH. Biological cross-talk between WNK1 and the transforming growth factor beta-Smad signaling pathway. *J Biol Chem.* 2007;282(25):17985-17996.
157. Couzens AL, Knight JD, Kean MJ, et al. Protein interaction network of the mammalian Hippo pathway reveals mechanisms of kinase-phosphatase interactions. *Sci Signal.* 2013;6(302):rs15.
158. Shi-wen X, Stanton LA, Kennedy L, et al. CCN2 is necessary for adhesive responses to transforming growth factor-beta1 in embryonic fibroblasts. *J Biol Chem.* 2006;281(16):10715-10726.
159. Unruh A, Ressel A, Mohamed HG, et al. The hypoxia-inducible factor-1 alpha is a negative factor for tumor therapy. *Oncogene.* 2003;22(21):3213-3220.
160. Feix G, Hake H. Primer directed initiation of RNA synthesis catalysed by Qbeta replicase. *Biochem Biophys Res Commun.* 1975;65(2):503-509.
161. Li G, Ci W, Karmakar S, et al. SPOP promotes tumorigenesis by acting as a key regulatory hub in kidney cancer. *Cancer Cell.* 2014;25(4):455-468.
162. Chin BY, Jiang G, Wegiel B, et al. Hypoxia-inducible factor 1alpha stabilization by carbon monoxide results in cytoprotective preconditioning. *Proc Natl Acad Sci U S A.* 2007;104(12):5109-5114.
163. Gorlach A, Diebold I, Schini-Kerth VB, et al. Thrombin activates the hypoxia-inducible factor-1 signaling pathway in vascular smooth muscle cells: Role of the p22(phox)-containing NADPH oxidase. *Circ Res.* 2001;89(1):47-54.
164. Elser M, Borsig L, Hassa PO, et al. Poly(ADP-ribose) polymerase 1 promotes tumor cell survival by coactivating hypoxia-inducible factor-1-dependent gene expression. *Mol Cancer Res.* 2008;6(2):282-290.
165. Sun X, Wei L, Chen Q, Terek RM. CXCR4/SDF1 mediate hypoxia induced chondrosarcoma cell invasion through ERK signaling and increased MMP1 expression. *Mol Cancer.* 2010;9:17.
166. Li G, Zhang Y, Qian Y, et al. Interleukin-17A promotes rheumatoid arthritis synoviocytes migration and invasion under hypoxia by increasing MMP2 and MMP9 expression through NF-kappaB/HIF-1alpha pathway. *Mol Immunol.* 2013;53(3):227-236.
167. Wang V, Davis DA, Haque M, Huang LE, Yarchoan R. Differential gene up-regulation by hypoxia-inducible factor-1alpha and hypoxia-inducible factor-2alpha in HEK293T cells. *Cancer Res.* 2005;65(8):3299-3306.
168. Liu Y, Hu Y, Li X, Niu L, Teng M. The crystal structure of the human nascent polypeptide-associated complex domain reveals a nucleic acid-binding region on the NACA subunit. *Biochemistry.* 2010;49(13):2890-2896.
169. Meury T, Akhouayri O, Jafarov T, Mandic V, St-Arnaud R. Nuclear alpha NAC influences bone matrix mineralization and osteoblast maturation in vivo. *Mol Cell Biol.* 2010;30(1):43-53.
170. Mendez MG, Restle D, Janmey PA. Vimentin enhances cell elastic behavior and protects against compressive stress. *Biophys J.* 2014;107(2):314-323.
171. Tilleman K, Van Steendam K, Cantaert T, De Keyser F, Elewaut D, Deforce D. Synovial detection and autoantibody reactivity of processed citrullinated isoforms of vimentin in inflammatory arthritides. *Rheumatology (Oxford).* 2008;47(5):597-604.

172. Cheng F, Shen Y, Mohanasundaram P, et al. Vimentin coordinates fibroblast proliferation and keratinocyte differentiation in wound healing via TGF-beta-Slug signaling. *Proc Natl Acad Sci U S A.* 2016;113(30):E4320-4327.
173. Qian L, Huang Y, Spencer CI, et al. In vivo reprogramming of murine cardiac fibroblasts into induced cardiomyocytes. *Nature.* 2012;485(7400):593-598.
174. Hsia LT, Ashley N, Ouaret D, Wang LM, Wilding J, Bodmer WF. Myofibroblasts are distinguished from activated skin fibroblasts by the expression of AOC3 and other associated markers. *Proc Natl Acad Sci U S A.* 2016;113(15):E2162-2171.
175. Nam YJ, Song K, Luo X, et al. Reprogramming of human fibroblasts toward a cardiac fate. *Proc Natl Acad Sci U S A.* 2013;110(14):5588-5593.
176. Kim H, Nakamura F, Lee W, Hong C, Perez-Sala D, McCulloch CA. Regulation of cell adhesion to collagen via beta1 integrins is dependent on interactions of filamin A with vimentin and protein kinase C epsilon. *Exp Cell Res.* 2010;316(11):1829-1844.
177. Hayashida T, Jones JC, Lee CK, Schnaper HW. Loss of beta1-integrin enhances TGF-beta1-induced collagen expression in epithelial cells via increased alphavbeta3-integrin and Rac1 activity. *J Biol Chem.* 2010;285(40):30741-30751.
178. Sudhakar A, Sugimoto H, Yang C, Lively J, Zeisberg M, Kalluri R. Human tumstatin and human endostatin exhibit distinct antiangiogenic activities mediated by alpha v beta 3 and alpha 5 beta 1 integrins. *Proc Natl Acad Sci U S A.* 2003;100(8):4766-4771.
179. Jo M, Eastman BM, Webb DL, Stoletov K, Klemke R, Gonias SL. Cell signaling by urokinase-type plasminogen activator receptor induces stem cell-like properties in breast cancer cells. *Cancer Res.* 2010;70(21):8948-8958.
180. Budatha M, Roshanravan S, Zheng Q, et al. Extracellular matrix proteases contribute to progression of pelvic organ prolapse in mice and humans. *J Clin Invest.* 2011;121(5):2048-2059.
181. Li S, Harrison D, Carbonetto S, et al. Matrix assembly, regulation, and survival functions of laminin and its receptors in embryonic stem cell differentiation. *J Cell Biol.* 2002;157(7):1279-1290.
182. Huttlin EL, Bruckner RJ, Paulo JA, et al. Architecture of the human interactome defines protein communities and disease networks. *Nature.* 2017;545(7655):505-509.
183. Brionne TC, Tesseur I, Masliah E, Wyss-Coray T. Loss of TGF-beta 1 leads to increased neuronal cell death and microgliosis in mouse brain. *Neuron.* 2003;40(6):1133-1145.
184. Spessotto P, Cervi M, Mucignat MT, et al. beta 1 Integrin-dependent cell adhesion to EMILIN-1 is mediated by the gC1q domain. *J Biol Chem.* 2003;278(8):6160-6167.
185. Danussi C, Petrucco A, Wassermann B, et al. EMILIN1-alpha4/alpha9 integrin interaction inhibits dermal fibroblast and keratinocyte proliferation. *J Cell Biol.* 2011;195(1):131-145.
186. Fabbro C, de Gemmis P, Braghetta P, et al. Analysis of regulatory regions of Emilin1 gene and their combinatorial contribution to tissue-specific transcription. *J Biol Chem.* 2005;280(16):15749-15760.
187. Zanetti M, Braghetta P, Sabatelli P, et al. EMILIN-1 deficiency induces elastogenesis and vascular cell defects. *Mol Cell Biol.* 2004;24(2):638-650.
188. Rolland T, Tasan M, Charloteaux B, et al. A proteome-scale map of the human interactome network. *Cell.* 2014;159(5):1212-1226.
189. Randell A, Daneshtalab N. Elastin microfibril interface-located protein 1, transforming growth factor beta, and implications on cardiovascular complications. *J Am Soc Hypertens.* 2017;11(7):437-448.
190. Alikhani Z, Alikhani M, Boyd CM, Nagao K, Trackman PC, Graves DT. Advanced glycation end products enhance expression of pro-apoptotic genes and stimulate fibroblast apoptosis through cytoplasmic and mitochondrial pathways. *J Biol Chem.* 2005;280(13):12087-12095.
191. Chen YS, Wang XJ, Feng W, Hua KQ. Advanced glycation end products decrease collagen I levels in fibroblasts from the vaginal wall of patients with POP via the RAGE, MAPK and NF-kappaB pathways. *Int J Mol Med.* 2017;40(4):987-998.
192. Brune M, Muller M, Melino G, Bierhaus A, Schilling T, Nawroth PP. Depletion of the receptor for advanced glycation end products (RAGE) sensitizes towards apoptosis via p53 and p73 posttranslational regulation. *Oncogene.* 2013;32(11):1460-1468.
193. Chen X, Zhang L, Zhang IY, et al. RAGE expression in tumor-associated macrophages promotes angiogenesis in glioma. *Cancer Res.* 2014;74(24):7285-7297.
194. Zhou Z, Immel D, Xi CX, et al. Regulation of osteoclast function and bone mass by RAGE. *J Exp Med.* 2006;203(4):1067-1080.
195. Fujimoto N, Terlizzi J, Aho S, et al. Extracellular matrix protein 1 inhibits the activity of matrix metalloproteinase 9 through high-affinity protein/protein interactions. *Exp Dermatol.* 2006;15(4):300-307.
196. Reiniger N, Lau K, McCalla D, et al. Deletion of the receptor for advanced glycation end products reduces glomerulosclerosis and preserves renal function in the diabetic OVE26 mouse. *Diabetes.* 2010;59(8):2043-2054.

197. Box JM, Kaur J, Stuart RA. MrpL35, a mitospecific component of mitoribosomes, plays a key role in cytochrome c oxidase assembly. *Mol Biol Cell.* 2017;28(24):3489-3499.
198. Alujevic Jakus I, Jakus D, Marinovic J, Cavar M, Banic I, Vilovic K. Expression of Mitochondrial Respiratory Chain Complexes in the Vaginal Wall in Postmenopausal Women with Pelvic Organ Prolapse. *Gynecol Obstet Invest.* 2018;83(5):487-492.
