# Supplementary material for: Molecular Landscape of Pelvic Organ Prolapse Provides Insights into Disease Etiology
Source: Int J Mol Sci. 2023 Mar 23;24(7):6087. doi: 10.3390/ijms24076087 (PMC10094264; doi:10.3390/ijms24076087)
Supplement: Supplementary file 1 [file ijms-24-06087-s001.zip › ijms-2158288-supplementary/Supplementary Table S1.pdf]

| **Supplementary Table S1.** Forty one candidate genes/molecules and two microRNAs (miRNAs) that are implicated in POP etiology through other evidence than our exome chip study, i.e., genetic association and/or mutation studies, miRNA/mRNA/protein expression studies and/or genetic animal model studies. | | |
|---|---|---|
| **Gene/miRNA/molecule** | **Evidence for involvement in POP etiology** | **Level of evidence *** |
| *BMP1* | BMP1 expression was significantly upregulated in the vaginal wall of patients with POP compared to healthy controls, and this difference was seen regardless of menopausal status[1]. | + |
| *CASP3* | CASP3 expression was found to be significantly increased in the uterosacral ligaments (USLs) of women with POP compared to healthy controls[2]. | + |
| *CDH1* | CDH1 expression was found to be significantly increased in vaginal cells of premenopausal women with POP compared to healthy controls[3]. | + |
| *COL1A1* | A meta-analysis of genetic studies indicated that rs1800012 in *COL1A1* is associated with POP[4]; the expression of COL1A1 was found to be different in USLs and in the anterior vaginal wall of women with POP compared to healthy controls[5]. | +++ |
| *COL3A1* | While several individual studies reported associations of SNPs within this gene with POP[6,7], a meta-analysis could not replicate this[4]; the expression of COL3A1 was found to be different in the anterior vaginal wall of women with POP compared to healthy controls[5]. | +++ |
| *COL18A1* | A GWAS showed that rs2236479 in *COL18A1* was significantly associated with POP[8] | + |
| *ECM1* | In a case-control study, ECM1 gene expression was found to be upregulated in women with POP[9]. | + |
| *ELN* | A decreased expression of ELN fibers in the anterior vaginal wall and USLs[5] of POP patients was reported; conversely, an increased ELN expression in the anterior vaginal wall of POP patients was also reported[10,11]. | +++ |
| *ESR1* | In a case-control study, rs2228480 in *ESR1* was found to be significantly associated with POP[12]. | + |
| *ESR2* | A haplotype of five SNPs in *ESR2* is associated with POP[13]; ESR2 expression was found to be significantly decreased in women with POP compared to healthy controls[5,14]. | +++ |

| Supplementary Table 1 - continued. | | |
|---|---|---|
| **Gene/miRNA/molecule** | **Evidence for involvement in POP etiology** | **Level of evidence *** |
| *FBLN3* | A GWAS showed that two SNPS in *FBLN3* (rs1430191, located 287 kb downstream and rs3791675, intronic) are associated at genome-wide significant level (P < 5,00E-08) with POP[15]. Female *Fbln3* knockout mice also show failure of pelvic organ support[16]. | ++ |
| *FBLN5* [a] | Two SNPS in *FBLN5* (rs2018736 and rs12589592) are significantly associated with POP[17]; a decreased expression of FBLN5 protein in the USLs, cardinal ligaments and in the pubocervical fascia[9] of patients with POP was reported, whereas an increased expression of FBLN5 mRNA in the USLs[5] and in the uterine cervix[11] of POP patients was found; female *Fbln5* knockout mice develop POP, which increases progressively with age and after delivery[18]. FBLN5 gene expression in USLs from women with POP increased with severity of POP[11]. | +++ |
| *GSTP1* | Ile105Val, a missense mutation in *GSTP1*, is a *protective* factor against advanced POP[19]. | + |
| *GPX1* | GPX1 expression was found to be significantly decreased in the pelvic support structures of women with POP compared to healthy controls[5]. In a case-control study, GPX1 protein content and enzymatic activity was found to be downregulated in the cardinal ligaments of women with POP[20]. | ++ |
| *HIF1A* | In a case-control study, fibroblasts from anterior vaginal wall tissues from postmenopausal women with POP had more HIF-1α expressed in the nucleus when compared to healthy controls[21]. | + |
| *HLA-DQA1* | HLA-DQA1 expression was found to be significantly increased in the uterosacral and round ligaments of women with POP compared to healthy controls[5]. | + |
| *HLA-DQB1* | HLA-DQB1 expression was found to be significantly increased in the uterosacral and round ligaments of women with POP compared to healthy controls[5]. | + |
| *ITGB1* | ITGB1 expression in uterosacral ligaments from women with POP increased with disease severity[11]. | + |

| Supplementary Table 1 - continued. | | |
|---|---|---|
| **Gene/miRNA/molecule** | **Evidence for involvement in POP etiology** | **Level of evidence *** |
| *LAMC1* | A variant in the promoter region of *LAMC1* gene (rs10911193) has been associated with familial POP[22], but this is not the case for women with POP in the general population[23]. | + |
| *LOX* | The promoter region of *LOX* was found to contain 66 methylated CG sites in women with POP versus only one methylated CG site in healthy controls, which indicates that methylation in the promoter region may specifically suppress LOX gene expression in women with POP[24]; the expression of LOX mRNA in the anterior vaginal wall and USLs[5] of POP patients was found to be significantly decreased; LOX expression was also found to be significantly decreased in vaginal cells of premenopausal women with POP compared to healthy controls[3]. | +++ |
| *LOXL1* | The expression of LOXL1 protein was found to be significantly decreased in the USLs, cardinal ligaments and anterior vaginal wall[5] of women with POP compared to healthy controls; a significantly decreased expression of LOXL1 mRNA in the anterior vaginal wall and USLs[5] of women with POP was reported; LOXL1 expression was also found to be significantly decreased in vaginal cells of premenopausal women with POP compared to healthy controls[3]; LOXL1 gene expression in USLs from women with POP increased with disease severity[11]; female *Loxl1*-deficient mice develop POP[25]. | +++ |
| *miR-221* | The expression of miR-221 was found to be significantly increased in the USLs of women with POP compared to healthy controls[5]. | + |
| *miR-222* | The expression of miR-222 was found to be significantly increased in the USLs of women with POP compared to healthy controls[5]. | + |
| *MMP1* | A SNP in *MMP1* was reported to be significantly associated with POP[26]. In addition, the combination of SNPS in *MMP1* and *MMP3* was found to be associated with POP, indicating genetic interaction - or epistasis - between *MMP1* and *MMP3*[27]; MMP1 expression was found to be significantly increased in the vaginal mucosa and USLs[5] of women with POP compared to healthy controls. | +++ |

| **Supplementary Table 1 - continued.** | | |
|---|---|---|
| **Gene/miRNA/molecule** | **Evidence for involvement in POP etiology** | **Level of evidence *** |
| *MMP2* | MMP2 expression was found to be significantly increased in the USLs and anterior vaginal wall[5] of women with POP compared to healthy controls; MMP2 was found to be differentially expressed in fibroblasts from the prolapsed site of women with POP[28]; MMP2 expression was also found to be significantly decreased in vaginal cells of premenopausal women with POP compared to healthy controls[3]. | +++ |
| *MMP3* | The combination of SNPS in *MMP1* and *MMP3* was found to be associated with POP, indicating genetic interaction - or epistasis - between *MMP1* and *MMP3*[27]; MMP3 expression was found to be significantly increased in the anterior vaginal wall of women with POP compared to healthy controls[5,12]; MMP3 expression was also found to be significantly increased in vaginal cells of premenopausal women with POP compared to healthy controls[3]. | +++ |
| *MMP8* | In a case-control study, MMP8 gene and protein expression were upregulated in vaginal tissue from women with severe POP (POP-Q ≥III) compared to women with mild POP (POP-Q=2)[5]. | + |
| *MMP9* | In a case-control study, rs3918253 and rs3918256 in *MMP9* were found to be significantly associated with POP[29]; MMP9 expression was found to be significantly increased in the anterior vaginal wall of women with POP compared to healthy controls [5]. | +++ |
| *NOP56* | A GWAS showed that rs1810636, a SNP 16 kb downstream of NOP56, was significantly associated with POP[8]. | + |
| *PARP1* | Val762Ala, a missense mutation in *PARP1*, is associated with a *decreased* risk of advanced POP[30]. | + |
| *PLAUR* [b] | PLAUR expression was found to be significantly increased in the uterosacral and round ligaments of women with POP compared to healthy controls[5]. | + |
| *PR* | In a case-control study, rs484389 in *PR* was found to be significantly associated with POP[31]; PR expression was found to be significantly decreased in the USLs of *post*menopausal women with POP while PR expression in the cardinal ligaments was reported to be significantly increased in *pre*menopausal women with POP[5]. | +++ |

| Supplementary Table 1 - continued. | | |
|---|---|---|
| **Gene/miRNA/molecule** | **Evidence for involvement in POP etiology** | **Level of evidence *** |
| *RAGE/AGE complex* | Advanced Glycation End (AGE) products have been found to be increased in the vaginal-epithelial and muscularis tissues from pre-menopausal women with POP[32,33]. In vitro studies have shown that AGEs inhibit the proliferation of and decreases collagen I expression in human vaginal fibroblasts from women with POP through signaling involving the receptor of AGEs (RAGE)[34]. | +++ |
| *SERPINA1* | SERPINA1 mRNA was found to be significantly downregulated in vaginal tissue[5,35] and USLs[5] of women with POP compared to healthy controls. | +++ |
| *SMAD3* | In a case control study, Smad-3 and phosphorylated Smad-3 were higher in the muscularis layer of vaginal tissues from premenopausal women with POP[33]. | + |
| *TBX5* | A GWAS showed that rs1247943, a SNP that is located 118 kb downstream from *TBX5*, was associated with POP at genome-wide significant level [15]. | + |
| *TGFB1* | In a case-control study, a significant negative correlation between POP-Q stage and the expression of TGFB1 in the pubocervical fascia was found[5]. In a second case-control study, a similar negative correlation between POP-Q stage and expression of TGFB1 in vaginal fibroblasts was reported[36]. | ++ |
| *TIMP1* | In a case-control study, TIMP1 gene and protein expression were downregulated in vaginal tissue from women with severe POP (POP-Q ≥III) compared to women with mild POP (POP-Q=2) and to controls[5]. | + |
| *TIMP2* | TIMP2 expression was found to be significantly decreased in the USLs of women with POP compared to healthy controls[5]; the expression of TIMP2 was also found to be different in vaginal cells of premenopausal women with POP compared to healthy controls[3]. | +++ |
| *TNFSF10* | The expression of TNFSF10 (other name: TRAIL) was found to be significantly increased in the uterosacral and round ligaments of women with POP compared to healthy controls[5]. | + |

| Supplementary Table 1 - continued. | | |
|---|---|---|
| **Gene/miRNA/molecule** | **Evidence for involvement in POP etiology** | **Level of evidence *** |
| *VIM* [c] | A case-control study found incresaed vimentin (VIM) in the cytoskeleton of cells from the vaginal wall and the USLs in women with POP[37]. | + |
| *WNK1* | A whole exome sequencing study identified multiple mutations in *WNK1* in women with POP[38]. | + |
| *WNT4* | A GWAS showed that rs3820282, an intronic SNP in *WNT4*, was associated at genome-wide significant level with POP[15]. | + |

Abbreviations: GWAS, genome-wide association study; POP, pelvic organ prolapse; POP-Q, Pelvic Organ Prolapse Quantification system; SNP(s), single nucleotide polymorphism(s); USLs, uterosacral ligaments.

***** The level of evidence for the involvement of each gene and/or its encoded protein in POP etiology is indicated as follows: '+', implicated in POP through a single genetic association/mutation study, miRNA/mRNA/protein expression study or genetic animal model study ; **'**++**'** implicated in POP through 2 independent genetic association/mutation studies, miRNA/mRNA/protein expression studies and/or genetic animal model studies ; '+++', implicated in POP through at least 3 independent genetic association/mutation studies, miRNA/mRNA/protein expression studies and/or genetic animal model studies.

Within our set of differentially expressed genes in an independent POP cohort (see Methods), the expression of three of the 40 additional genes from the landscape was different: the expression of *FBLN5* was downregulated (FC= -1,41 ; corrected P=8.56E-03) [a.], the expression of *PLAUR* was upregulated (FC= 1,61 ; corrected P=2.25E-02) [b.], and the expression of *VIM* was upregulated (FC= 1,20 ; corrected P=3.59E-02) [c.].

**References**


1. Borazjani A, Kow N, Harris S, Ridgeway B, Damaser MS. Transcriptional Regulation of Connective Tissue Metabolism Genes in Women With Pelvic Organ Prolapse. *Female Pelvic Med Reconstr Surg.* 2017;23(1):44-52.
2. Kim EJ, Chung N, Park SH, et al. Involvement of oxidative stress and mitochondrial apoptosis in the pathogenesis of pelvic organ prolapse. *J Urol.* 2013;189(2):588-594.
3. Kufaishi H, Alarab M, Drutz H, Lye S, Shynlova O. Comparative Characterization of Vaginal Cells Derived From Premenopausal Women With and Without Severe Pelvic Organ Prolapse. *Reprod Sci.* 2016;23(7):931-943.
4. Cartwright R, Kirby AC, Tikkinen KA, et al. Systematic review and metaanalysis of genetic association studies of urinary symptoms and prolapse in women. *Am J Obstet Gynecol.* 2015;212(2):199 e191-124.
5. Khadzhieva MB, Kolobkov DS, Kamoeva SV, Salnikova LE. Expression changes in pelvic organ prolapse: a systematic review and in silico study. *Sci Rep.* 2017;7(1):7668.
6. Chen HY, Chung YW, Lin WY, Wang JC, Tsai FJ, Tsai CH. Collagen type 3 alpha 1 polymorphism and risk of pelvic organ prolapse. *Int J Gynaecol Obstet.* 2008;103(1):55-58.
7. Jeon MJ, Chung SM, Choi JR, Jung HJ, Kim SK, Bai SW. The relationship between COL3A1 exon 31 polymorphism and pelvic organ prolapse. *J Urol.* 2009;181(3):1213-1216.
8. Allen-Brady K, Cannon-Albright L, Farnham JM, et al. Identification of six loci associated with pelvic organ prolapse using genome-wide association analysis. *Obstet Gynecol.* 2011;118(6):1345-1353.
9. Cecati M, Corradetti A, Sartini D, et al. Expression of extracellular matrix and adhesion proteins in pelvic organ prolapse. *Cell Mol Biol (Noisy-le-grand).* 2018;64(5):142-148.
10. Zong W, Stein SE, Starcher B, Meyn LA, Moalli PA. Alteration of vaginal elastin metabolism in women with pelvic organ prolapse. *Obstet Gynecol.* 2010;115(5):953-961.
11. Wang H, Kira Y, Hamuro A, Takase A, Tachibana D, Koyama M. Differential gene expression of extracellular-matrix-related proteins in the vaginal apical compartment of women with pelvic organ prolapse. *Int Urogynecol J.* 2018.
12. Chen HY, Chung YW, Lin WY, Chen WC, Tsai FJ, Tsai CH. Estrogen receptor alpha polymorphism is associated with pelvic organ prolapse risk. *Int Urogynecol J Pelvic Floor Dysfunct.* 2008;19(8):1159-1163.
13. Chen HY, Wan L, Chung YW, Chen WC, Tsai FJ, Tsai CH. Estrogen receptor beta gene haplotype is associated with pelvic organ prolapse. *Eur J Obstet Gynecol Reprod Biol.* 2008;138(1):105-109.
14. Skala CE, Petry IB, Albrich S, Puhl A, Naumann G, Koelbl H. The effect of genital and lower urinary tract symptoms on steroid receptor expression in women with genital prolapse. *Int Urogynecol J.* 2011;22(6):705-712.
15. Olafsdottir T, Thorleifsson G, Sulem P, et al. Genome-wide association identifies seven loci for pelvic organ prolapse in Iceland and the UK Biobank. *Commun Biol.* 2020;3(1):129.
16. Rahn DD, Acevedo JF, Roshanravan S, et al. Failure of pelvic organ support in mice deficient in fibulin-3. *Am J Pathol.* 2009;174(1):206-215.
17. Khadzhieva MB, Kamoeva SV, Chumachenko AG, et al. Fibulin-5 (FBLN5) gene polymorphism is associated with pelvic organ prolapse. *Maturitas.* 2014;78(4):287-292.
18. Drewes PG, Yanagisawa H, Starcher B, et al. Pelvic organ prolapse in fibulin-5 knockout mice: pregnancy-induced changes in elastic fiber homeostasis in mouse vagina. *Am J Pathol.* 2007;170(2):578-589.
19. Kim JY, Kim EJ, Jeon MJ, Kim R, Lee MW, Kim SW. Association between susceptibility to advanced pelvic organ prolapse and glutathione S-transferase P1 Ile105Val polymorphism. *Eur J Obstet Gynecol Reprod Biol.* 2014;175:205-208.
20. Fang G, Hong L, Liu C, et al. Oxidative status of cardinal ligament in pelvic organ prolapse. *Exp Ther Med.* 2018;16(4):3293-3302.
21. Jakus IA, Jakus D, Aracic N, Stipic I, Vilovic K. Immunohistochemical expression of hypoxia-inducible factor-1alpha in stromal cells of vaginal tissue in post-menopausal women with pelvic organ prolapse. *Indian J Med Res.* 2017;146(Suppl):S63-S67.

22. Nikolova G, Lee H, Berkovitz S, et al. Sequence variant in the laminin gamma1 (LAMC1) gene associated with familial pelvic organ prolapse. *Hum Genet.* 2007;120(6):847-856.
23. Nakad B, Fares F, Azzam N, Feiner B, Zilberlicht A, Abramov Y. Estrogen receptor and laminin genetic polymorphism among women with pelvic organ prolapse. *Taiwan J Obstet Gynecol.* 2017;56(6):750-754.
24. Klutke J, Stanczyk FZ, Ji Q, Campeau JD, Klutke CG. Suppression of lysyl oxidase gene expression by methylation in pelvic organ prolapse. *Int Urogynecol J.* 2010;21(7):869-872.
25. Liu X, Zhao Y, Gao J, et al. Elastic fiber homeostasis requires lysyl oxidase-like 1 protein. *Nat Genet.* 2004;36(2):178-182.
26. Ferrari MM, Rossi G, Biondi ML, Vigano P, Dell'utri C, Meschia M. Type I collagen and matrix metalloproteinase 1, 3 and 9 gene polymorphisms in the predisposition to pelvic organ prolapse. *Arch Gynecol Obstet.* 2012;285(6):1581-1586.
27. Skorupski P, Jankiewicz K, Miotla P, Marczak M, Kulik-Rechberger B, Rechberger T. The polymorphisms of the MMP-1 and the MMP-3 genes and the risk of pelvic organ prolapse. *Int Urogynecol J.* 2013;24(6):1033-1038.
28. Ruiz-Zapata AM, Kerkhof MH, Zandieh-Doulabi B, Brolmann HA, Smit TH, Helder MN. Functional characteristics of vaginal fibroblastic cells from premenopausal women with pelvic organ prolapse. *Mol Hum Reprod.* 2014;20(11):1135-1143.
29. Wu JM, Visco AG, Grass EA, et al. Matrix metalloproteinase-9 genetic polymorphisms and the risk for advanced pelvic organ prolapse. *Obstet Gynecol.* 2012;120(3):587-593.
30. Kim JY, Kim EJ, Jeon MJ, Kim H, Moon YJ, Bai SW. Association between the poly(ADP-ribose) polymerase-1 gene polymorphism and advanced pelvic organ prolapse. *Menopause.* 2014;21(2):177-181.
31. Chen HY, Chung YW, Lin WY, Chen WC, Tsai FJ, Tsai CH. Progesterone receptor polymorphism is associated with pelvic organ prolapse risk. *Acta Obstet Gynecol Scand.* 2009;88(7):835-838.
32. Jackson SR, Avery NC, Tarlton JF, Eckford SD, Abrams P, Bailey AJ. Changes in metabolism of collagen in genitourinary prolapse. *Lancet.* 1996;347(9016):1658-1661.
33. Vetuschi A, Pompili S, Gallone A, et al. Immunolocalization of Advanced Glycation End Products, Mitogen Activated Protein Kinases, and Transforming Growth Factor-beta/Smads in Pelvic Organ Prolapse. *J Histochem Cytochem.* 2018;66(9):673-686.
34. Chen YS, Wang XJ, Feng W, Hua KQ. Advanced glycation end products decrease collagen I levels in fibroblasts from the vaginal wall of patients with POP via the RAGE, MAPK and NF-kappaB pathways. *Int J Mol Med.* 2017;40(4):987-998.
35. Chen B, Wen Y, Polan ML. Elastolytic activity in women with stress urinary incontinence and pelvic organ prolapse. *Neurourol Urodyn.* 2004;23(2):119-126.
36. Qi XY, Hong L, Guo FQ, Fu Q, Chen L, Li BS. Expression of transforming growth factor-beta 1 and connective tissue growth factor in women with pelvic organ prolapse. *Saudi Med J.* 2011;32(5):474-478.
37. Zeng C, Liu J, Wang H, Zhou Y, Wu J, Yan G. Correlation Between Autophagy and Collagen Deposition in Patients With Pelvic Organ Prolapse. *Female Pelvic Med Reconstr Surg.* 2018;24(3):213-221.
38. Rao S, Lang J, Zhu L, Chen J. Exome sequencing identifies a novel gene, WNK1, for susceptibility to pelvic organ prolapse (POP). *PLoS One.* 2015;10(3):e0119482.
