# Supplementary material for: Molecular Landscape of Pelvic Organ Prolapse Provides Insights into Disease Etiology
Source: Int J Mol Sci. 2023 Mar 23;24(7):6087. doi: 10.3390/ijms24076087 (PMC10094264; doi:10.3390/ijms24076087)
Supplement: Supplementary file 1 [file ijms-24-06087-s001.zip › ijms-2158288-supplementary/Supplementary Table S5.pdf]

**Supplementary Table S5.** Primer sequences used for the RT-PCR of the in vitro model showing the effect of metformin on fibroblasts from women with POP (**Figure 2**).				
**Target gene**		**Oligonucleotide sequence**	**Annealing temperature (°C)**	**Product Size (bp)**
COL1A1	Forward<br>Reverse	5’ TCCAACGAGATCGAGATCC 3’<br>5’ AAGCCGAATTCCTGGTCT 3’	56.05<br>55.76	191
COL3A1	Forward<br>Reverse	5’ GATCCGTTCTCTGCGATGAC 3’<br>5’ AGTTCTGAGGACCAGTAGGG 3’	58.52<br>57.76	279
ELN	Forward<br>Reverse	5' TTTGGCCCGGGAGTAGTTGG 3'<br>5' CAGCTGCTTCTGGTGACACAAC 3'	62.13<br>62.2	136
YWHAZ	Forward<br>Reverse	5’ GATGAAGCCATTGCTGAACTTG 3’<br>5’ CTATTTGTGGGACAGCATGGA 3’	58.49<br>58.00	229
HPRT1	Forward<br>Reverse	5’ GCTGACCTGCTGGATTACAT 3’<br>5’ CTTGCGACCTTGACCATCT 3’	57.66<br>57.46	260

RT-PCR, reverse-transcription polymerase chain reaction; COL1A1, α1(I)procollagen; COL3A1, α1(III)procollagen; ELN, elastin; YWHAZ, tyrosine 3-monooxygenase /tryptophan 5-monooxygenase activation protein, zeta polypeptide; HPRT1, hypoxanthine phosphoribosyltransferase 1.
